# Systematic mining and characterization of metal transporter families regulating zinc homeostasis provide insights into metal homeostasis in *Camellia sinensis*

**DOI:** 10.3389/fpls.2026.1901777

**Published:** 2026-08-18

**Authors:** Xin Zhang, Shuting Zheng, Li Zhang, Cheng Zhang, Huarong Tong, Lianyu Yuan

**Affiliations:** 1 College of Food Science, Southwest University, Chongqing, China; 2 Chongqing Agricultural Technology Extension Station, Chongqing, China; 3 Nanchuan District’s Agricultural Characteristic Industry Development Center of Chongqing Municipality, Chongqing, China

**Keywords:** biofortification, *Camellia sinensis*, gene family, metal transporter, zinc homeostasis

## Abstract

**Background and aims:**

Zinc is essential for tea plant growth and quality formation, yet its homeostatic mechanisms remain poorly understood. This study identified metal transporter families regulating zinc homeostasis, analyzed their evolution, structure, and expression, and clarified zinc uptake, transport, detoxification networks, and their links to metabolism.

**Methods:**

This study identified zinc homeostasis-related metal transporter families in the tea plant genome, characterized their structural features and expression profiles across tissues and developmental stages through integrative bioinformatics and transcriptomic analyses, and delineated the molecular mechanisms underlying zinc uptake, translocation, and detoxification by systematically integrating published evidence.

**Results:**

This study identified 74 metal transporter genes from six families: 13 *CsZIPs*, 12 *CsNRAMPs*, 10 *CsHMAs*, 10 *CsYSLs*, 14 *CsMTPs*, and 15 *CsCAXs* in the ‘Shuchazao2’ genome, revealing closer affinity to woody species than to *Arabidopsis*. These proteins exhibit conserved domains, diverse subcellular localizations (cell membrane, vacuole, chloroplast, and Golgi apparatus), and tissue-specific expression with abundant stress/hormone-responsive *cis*-elements. At the plant-soil interface, tea plants mobilize rhizospheric zinc via proton and organic acid secretion; CsYSLs, CsNRAMPs, and CsZIPs mediate zinc uptake, aided by arbuscular mycorrhizal fungi (AMF) and plant growth-promoting rhizobacteria (PGPR) that expand root absorption zones. Xylem CsHMAs and phloem CsYSLs coordinate root-to-shoot zinc translocation, and vacuolar transporters (CsMTPs, CsCAXs), cell wall immobilization, and antioxidant systems alleviate high-zinc stress injury.

**Conclusions:**

These findings collectively delineate an integrated zinc “acquisition-distribution-buffering” network in tea plants, offering a repertoire of candidate genes with potential utility in zinc biofortification breeding and improving acid soil adaptation. Further experimental validation, including tea transgenesis, zinc-stress qRT-PCR, and heterologous functional complementation, is essential to substantiate their biological roles.

## 1 Introduction

### 1.1 Physiological functions of zinc as an essential micronutrient in plants

As essential micronutrients and protein cofactors, metal ions such as Zn^2+^, Fe^2+^, Mn^2+^, and Cu^2+^ participate in fundamental physiological processes in plants (Hell and Stephan, 2003; Bozzi et al., 2016). However, metal excess exerts phytotoxicity, damaging root systems, impairing nutrient uptake, and inhibiting plant development (Alsafran et al., 2023). Meanwhile, escalating heavy metal contamination in agricultural soils poses severe threats to sustainable agricultural production (Hou et al., 2025). Conversely, metal deficiency suppresses seed germination and induces chromosomal aberrations (Sinclair and Krämer, 2012). Therefore, precise regulation of metal compartmentalization and concentration balance within cells is critical for maintaining metabolic homeostasis and adaptive plasticity under fluctuating soil availability (Kraemer, 2024). As a cofactor for enzymes and transcription factors, zinc is essential for plant metabolism, participating in DNA/RNA synthesis, hormone regulation, and photosystem repair (Prasad, 2012; Kolaj-Robin et al., 2015; Kraemer, 2025). Tea contains water-soluble zinc, offering dietary benefits (Palmgren et al., 2008); however, deficiency causes interveinal chlorosis and yield reduction, while toxicity (>200 mg·kg^-1^ soil) disrupts photosynthesis and membrane integrity (Kawachi et al., 2009; Broadley et al., 2012). Thus, regulating intracellular zinc homeostasis is crucial for plant performance (Choi et al., 2018).

### 1.2 The absorption, transport, and efflux of zinc in plants

Plants typically absorb Zn^2+^ via their roots, translocate it through the xylem, and ultimately sequester it in vacuoles. Root Zn^2+^ concentrations usually exceed those of above-ground tissues, with juvenile organs exhibiting higher zinc accumulation than mature ones (Jain et al., 2013). Soil zinc availability is closely associated with parent material, soil texture, and weathering intensity (Chesworth, 1991). In tea plants, zinc enhances nitrogen and phosphorus uptake by roots, thereby promoting bud sprouting and vigorous vegetative growth. Soil pH strongly influences zinc speciation. In acidic soils, zinc mainly exists as free ions with high bioavailability (Alloway, 2009). As acidophiles, tea plants are highly sensitive to soil pH. Although they thrive at pH 5.5, their zinc uptake declines sharply as soil pH rises (Li et al., 2023). Foliar zinc application significantly boosts tea yield and increases secondary metabolite concentrations. Therefore, balanced fertilization strategies that enhance soil nutrient availability and zinc accessibility are critical for improving tea quality and zinc content (Huang et al., 2022).

The molecular basis of how plants selectively absorb metal ions, maintain tissue homeostasis, and detoxify excess metals has emerged as a central research focus in plant nutrition (Meng et al., 2017). Over evolutionary time, plants have evolved sophisticated, finely tuned regulatory networks that orchestrate the uptake, efflux, chelation, detoxification, sequestration, and storage of metal ions, particularly Zn^2+^, across cells and organs, enabling them to adapt to environmental fluctuations and maintain nutritional balance (Clemens et al., 2002; Nevo and Nelson, 2006; Palmgren et al., 2008). Multiple membrane transport protein families form a layered homeostatic network that coordinates Zn^2+^ uptake, long-distance translocation, and intracellular sequestration (Kraemer, 2025). Broadly, they can be categorized into two functional groups: importers (uptake transporters) and effluxers/sequestrators (export and compartmentalization proteins). Importer families, including ZIP (ZRT/IRT-like proteins) (Ishimaru et al., 2007), YSL (Yellow-Stripe-Like proteins) (Waters et al., 2006), and NRAMP (Natural Resistance-Associated Macrophage Proteins), are localized at the plasma membrane (Oomen et al., 2009). They mediate the transport of divalent cations, including Zn^2+^, into the cytosol to sustain essential metabolic processes. Effluxer and sequestrator families are responsible for detoxifying excess cytoplasmic Zn^2+^ through active export or vacuolar compartmentalization. These include HMA (Heavy Metal ATPases, P1B-type ATPases), localized at the plasma membrane or tonoplast (Morel et al., 2009), which actively pump Zn^2+^ out of the cell or into vacuoles; MTP (Metal Tolerance Proteins, formerly designated CDF: cation diffusion facilitators), which mediate Zn^2+^ sequestration into vacuoles (Desbrosses-Fonrouge et al., 2005); and CAX (Ca^2+^/H^+^ exchanger antiporters) (Pittman et al., 2004), which primarily transport Ca^2+^ but also participate in Zn^2+^/Cd^2+^ translocation across the tonoplast (Hall and Williams, 2003).

### 1.3 Zinc homeostasis in tea plants

As the most widely consumed non-alcoholic beverage, tea is a rich source of essential micronutrients, including Zn, Mn, Fe, Cu, Mg, and K, which are vital for human health. Moderate daily consumption provides distinct physiological benefits (Nath Nath, 2012). Trace metals serve as hallmark signatures of tea’s intrinsic quality. Among them, zinc content is a key indicator used in grading green tea, making precise control of its level essential (Karak and Bhagat, 2010). Metal ion uptake and transport in plants are intricate, yet research has focused largely on herbaceous species. With faster auxin dynamics and greater biomass, woody plants, including tea, offer significant untapped potential for phytoremediation of heavy-metal contamination (Singh et al., 2016). Hence, the metal-transport machinery of tea plants must be elucidated. Thriving in acidic soils, tea plants readily mobilize and accumulate heavy metals that exist as labile ions or complexes in such environments. The publication of the Tea Plant Information Archive (TPIA) database has provided multiomics information on tea plants (Xia et al., 2019). To date, only a handful of *CsMTP*, *CsZIP,* and *CsNRAMP* genes have been characterized, and systematic investigations into the regulation and transport of metal ions, especially zinc, remain scarce (Zhang et al., 2020; Li et al., 2021; Zheng et al., 2022). This knowledge gap hinders both germplasm innovation and the targeted improvement of tea cultivars (Yuan et al., 2017).

Although the canonical pathways of plant zinc homeostasis have been elucidated in model species such as *Arabidopsis* and *Oryza sativa* (Huang et al., 2024; Kraemer, 2025), the tea plant (*Camellia sinensis*), as a typical acid-tolerant and aluminum-resistant woody cash crop, exhibits zinc nutritional mechanisms that are far more complex than those predicted by herbaceous model systems. In tea plants, zinc homeostasis operates as a dynamic system integrating rhizosphere microbiome signaling with quality-related metabolic networks. The following sections systematically review the molecular mechanisms underlying zinc acquisition, distribution, and regulation in tea plants, with particular emphasis on identifying transport proteins and elucidating the mechanisms that maintain zinc homeostatic balance. This work lays the foundation for understanding the mechanisms of Zn^2+^ absorption and transport in tea plants and provides a reference for tea genetic breeding, scientific fertilization, and the development of zinc-enriched tea products.

## 2 Materials and methods

### 2.1 Sequence acquisition and alignment

The protein sequences of the MTP, ZIP, HMA, CAX, NRAMP, and YSL families in *Vitis vinifera, Malus domestica*, and *Populus trichocarpa* were obtained from Phytozome 14 (https://phytozome-next.jgi.doe.gov/). The protein sequences of the AtMTP, AtZIP, AtHMA, AtCAX, AtNRAMP, and AtYSL families from *Arabidopsis thaliana* were retrieved from the *Arabidopsis* Information Resource (TAIR, https://www.arabidopsis.org/). Using these *Arabidopsis* protein sequences as query sequences, BLASTP searches were performed against the TPIA (https://tpia.teaplants.cn/) with an E-value threshold of 1e^-5^ to identify homologous genes belonging to the corresponding families in tea plants. The Pfam and CDD databases from NCBI (https://www.ncbi.nlm.nih.gov/) were used to verify the integrity of conserved domains in candidate proteins. The gene IDs, genomic sequences, CDS sequences, and protein sequences of the genes belonging to these six families in tea plants were downloaded from TPIA (Xia et al., 2019).

### 2.2 Physicochemical characteristics analysis

The physicochemical characteristics of proteins from each gene family in tea plants were analyzed using the ProtParam online tool (https://web.expasy.org/protparam/) provided by the ExPASy server, including amino acid length (aa), molecular weight (MW), isoelectric point (pI), and grand average of hydropathy (GRAVY). Subcellular localization prediction of these proteins was performed using the PlantmPLoc online platform (http://www.csbio.sjtu.edu.cn/bioinf/plant-multi/). Transmembrane domains (TMDs) were predicted and comparatively analyzed using the TMHMM-2.0 online server (https://services.healthtech.dtu.dk/services/TMHMM-2.0/) combined with DNAMAN software (Krogh et al., 2001). The tertiary structures of the proteins were constructed and predicted using the SWISS-MODEL database (https://swissmodel.expasy.org).

### 2.3 Multiple sequence alignments and phylogenetic tree construction

Multiple sequence alignments of the six family protein sequences from tea plants were performed using DNAMAN. Combined with the ClustalW program embedded in MEGA11 (Tamura et al., 2021), phylogenetic trees for the six protein families from tea plants and *Arabidopsis* were constructed via the neighbor-joining (NJ) method, with 1,000 bootstrap replicates. Gene nomenclature for the identified tea plant genes was determined according to their phylogenetic relationships and sequence similarities to the corresponding family members in *Arabidopsis*. To investigate the phylogenetic relationships of the six protein families between tea plants and other plant species, individual phylogenetic trees for each family were generated using the same approach. All resulting phylogenetic trees were visualized using the online platform iTOL (https://itol.embl.de/).

### 2.4 Gene structure, protein conserved motif, and *cis*-regulatory element analysis

Conserved motifs of six tea plant gene family proteins were detected using the online MEME tool (https://meme-suite.org/meme/tools/meme). Relevant parameters were configured with a maximum of 10 motifs and an optimal motif width of 6 to 60 amino acids (Bailey et al., 2015). Based on the GFF annotation files of the ‘Shuchazao2’ genome database, the exon-intron structural characteristics of the members from the six gene families were visualized and analyzed using the Gene Structure Display Server 2.0 (https://gsds.gao-lab.org/index.php) (Hu et al., 2015). Moreover, the 2-kb upstream promoter sequences of all six gene family members were extracted and submitted to the PlantCARE online database (http://bioinformatics.psb.ugent.be/webtools/plantcare/html/) for the prediction and analysis of *cis*-acting regulatory elements in the promoter regions.

### 2.5 Chromosomal location, gene duplication, and synteny analysis

TBtools-II software was used to analyze the chromosomal distribution characteristics of six gene families in tea plants based on the GFF3 annotation files of the ‘Shuchazao2’ genome. The MCScanX toolkit embedded in TBtools-II was used to identify intraspecies gene duplication events within tea plants, and the results were visualized using the Advanced Circos module. To elucidate the selective pressures acting on the duplicated gene pairs, the ratio of non-synonymous substitutions to synonymous substitutions (Ka/Ks) was calculated using the Simple Ka/Ks calculator tool. Furthermore, the MCScanX module was applied to detect cross-species syntenic blocks between tea plants and related species, including *Vitis vinifera*, *Arabidopsis*, *Malus domestica*, and *Populus trichocarpa*. The synteny analysis results were subsequently visualized using the Multiple Synteny Plot module in TBtools-II (Chen et al., 2020).

### 2.6 Gene expression analysis

Expression data for the six gene families in tea plants were retrieved from the TPIA, with TPM (Transcripts Per Million) as the quantitative unit of gene expression. Transcriptomic data for these genes in eight tissues (root, stem, bud, flower, fruit, young leaf, mature leaf, and old leaf) and at different leaf developmental stages (bud, the first leaf, the second leaf, the third leaf, and the fourth leaf below the bud) were downloaded, row-wise normalized, and visualized as a heatmap.

## 3 Results

**Figure 1:**
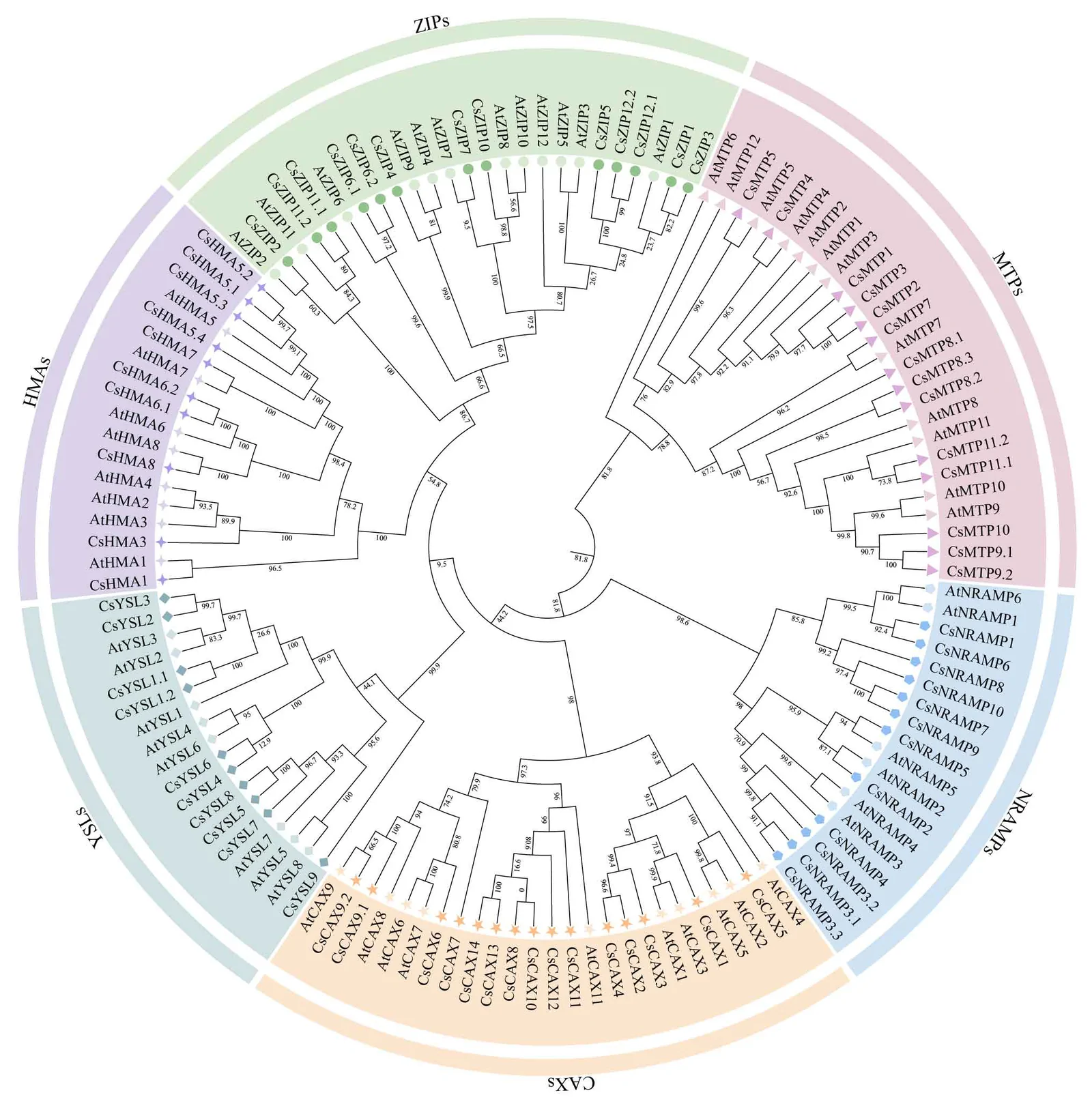
Phylogenetic relationship of CsZIP, CsNRAMP, CsHMA, CsYSL, CsMTP, and CsCAX family proteins in tea plants. The phylogenetic tree was constructed using the neighbor-joining (NJ) method in MEGA11 with 1000 bootstrap replicates. Different gene families are distinguished by distinct color-coded branches. The detailed information on the protein members used is provided in **Supplementary Table 1**.

The acquisition, translocation, and homeostatic maintenance of Zn^2+^ in tea plants operate as an intricate network requiring concerted action among diverse transporter families, regulatory modulators, and organellar compartments. We systematically delineated the underlying regulatory mechanisms from three core dimensions: root Zn^2+^ uptake, long-distance translocation, and intracellular zinc homeostasis. Six metal transporter families associated with zinc homeostasis–CsZIPs, CsNRAMPs, CsHMAs, CsYSLs, CsMTPs, and CsCAXs–comprising a total of 74 members (**Figure 1**), were identified from the tea plant genome and subjected to comprehensive bioinformatic and expression profiling analyses.

### 3.1 The CsZIP protein family in tea plants

**Figure 2:**
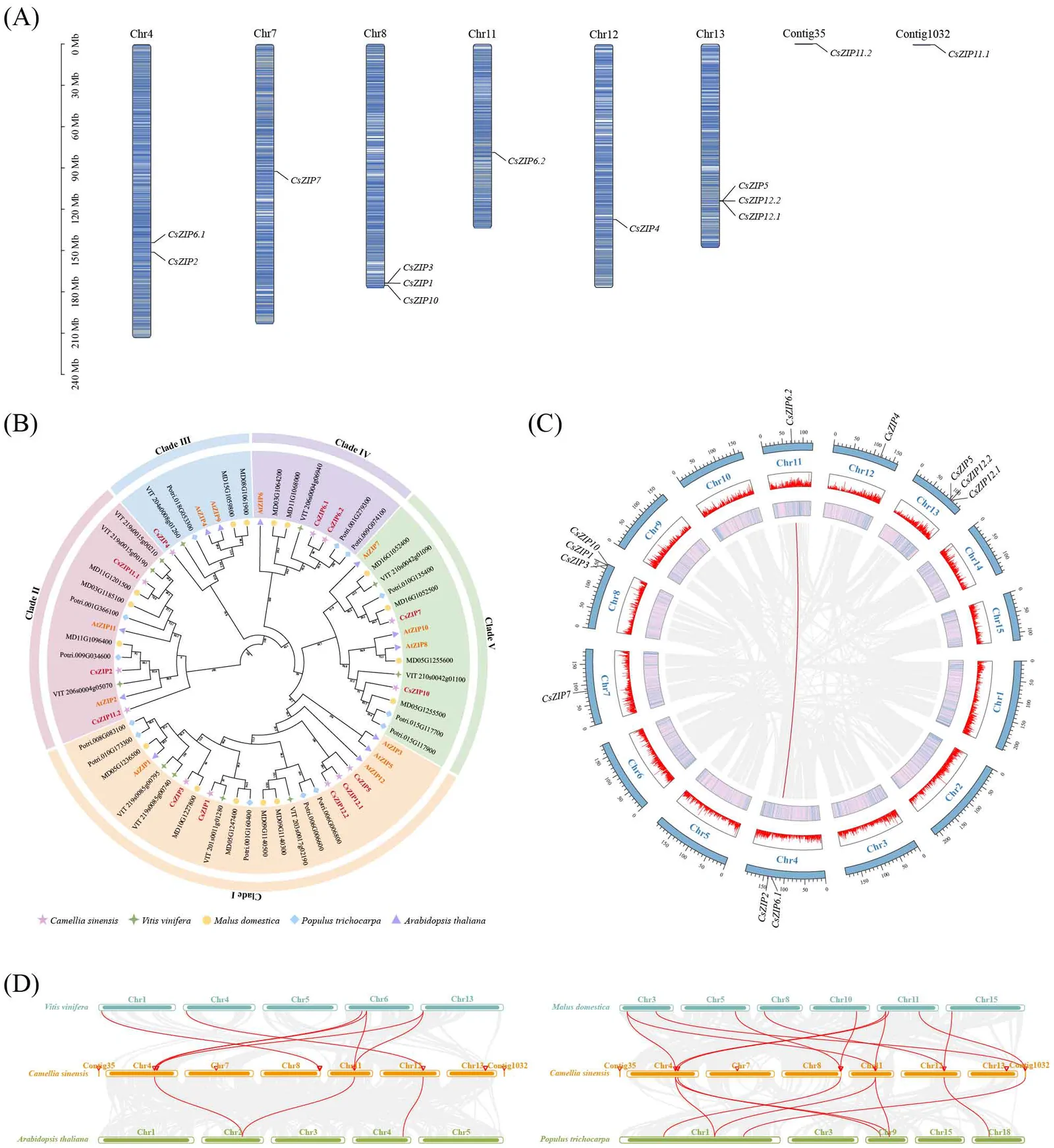
Chromosome distribution, phylogenetic relationship, and synteny analysis of *CsZIP* gene family. **(A)** Chromosomal localization of *CsZIPs* in the tea plant genome. **(B)** Phylogenetic relationship of ZIP family proteins from tea plants and other plants. The phylogenetic tree was constructed using the neighbor-joining (NJ) method in MEGA11 with 1000 bootstrap replicates. **(C)** Intraspecies collinearity analysis of *CsZIPs* in tea plants. Gray lines represent all collinear blocks within the tea genome, and red lines highlight duplicated *CsZIPs* gene pairs. **(D)** Interspecies collinearity analysis among tea plant, *Vitis vinifera*, *Arabidopsis*, *Populus trichocarpa*, and *Malus domestica*. Gray lines represent syntenic blocks across genomes, and red lines denote orthologous *CsZIP* relationships.

A total of 13 ZIP (ZRT/IRT-like proteins) family genes were identified in the tea plant genome, which primarily function in the uptake and transport of Zn^2+^. These genes were unevenly distributed on chromosomes 4, 7, 8, 11, 12, and 13. Among them, *CsZIP11.1* and *CsZIP11.2* were located on scaffold contig1032 and contig35, respectively (**Figure 2A**). Phylogenetic analysis classified CsZIP proteins into five subfamilies. Subfamily I contained the most members (five), whereas Subfamily III comprised only CsZIP4 (**Figure 2B**). Only one collinear gene pair (*CsZIP6.1*/*CsZIP6.2*) was identified within the tea genome, with a Ka/Ks value of 0.152 (<1), indicating that this gene pair has undergone purifying selection (**Figure 2C**; **Supplementary Table 1**). Interspecific collinearity analysis showed that tea plants shared the greatest number of ZIP collinear gene pairs with apple (11 pairs), followed by poplar (8 pairs) and grape (7 pairs), while *Arabidopsis* had the fewest (3 pairs). This suggests that the evolutionary pattern of the ZIP family in tea plants more closely resembles that of woody plants (**Figure 2D**).

**Figure 3:**
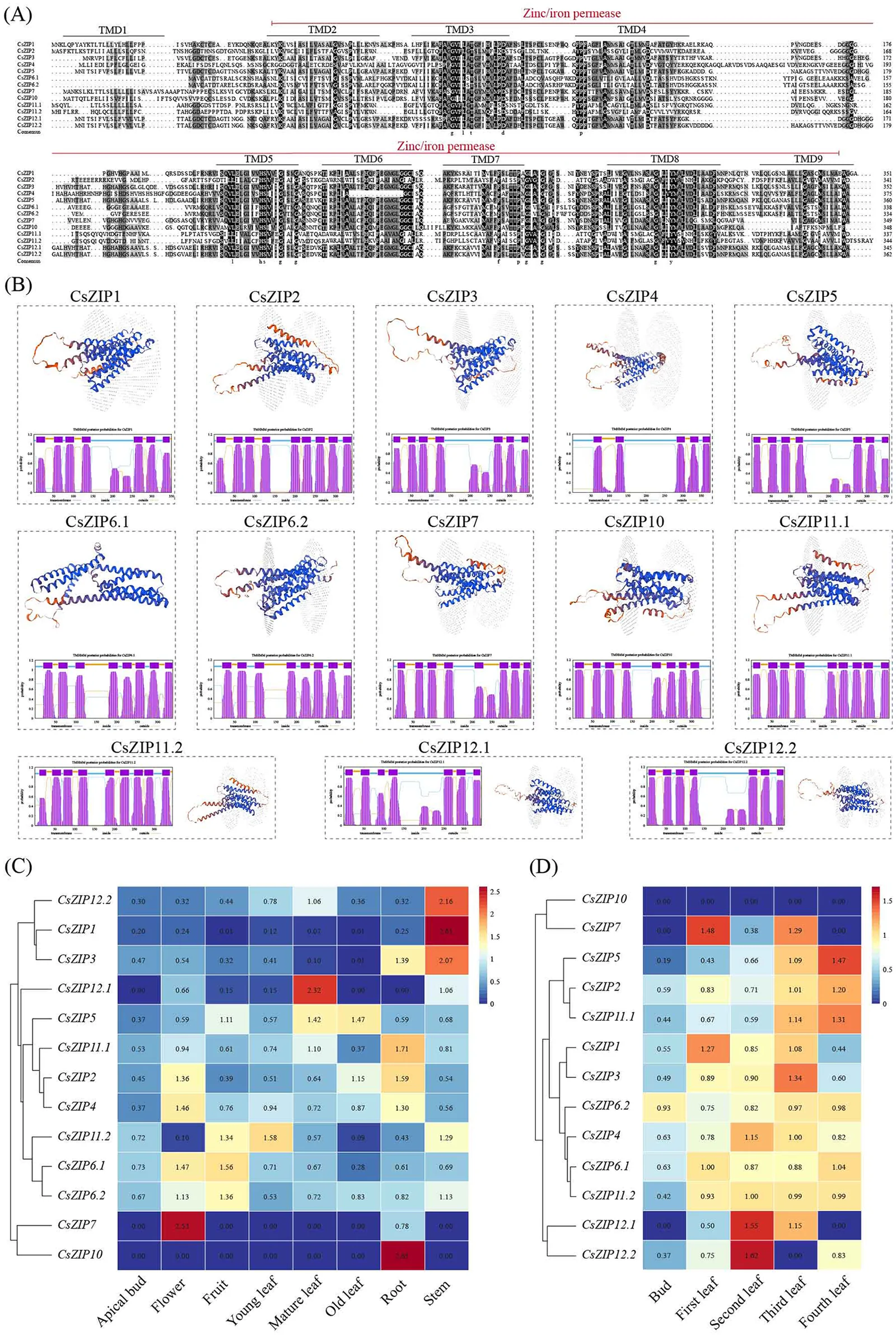
Multiple sequence alignment, three-dimensional structure modeling, transmembrane domain prediction, and tissue-specific expression profiling of CsZIPs in tea plants. **(A)** Multiple sequence alignment analysis of CsZIP proteins in tea plant. The black bars above the sequences denote the transmembrane domains (TMDs). The red bars above the sequences denote the Zinc/iron permease domain. Alignment coloring: black, ≥75% conservation; dark gray, ≥50% conservation; light gray, ≥33% conservation; dots, gaps. **(B)** Predicted transmembrane domains and three-dimensional structures of CsZIP proteins. The disk-shaped structure composed of gray dots in the three-dimensional protein models represents the membrane. In the transmembrane domain probability plots, purple peaks indicate transmembrane regions, the blue line corresponds to the intracellular compartment, and the yellow line corresponds to the extracellular compartment. **(C)** Heatmap of *CsZIPs* gene expression in apical bud, flower, fruit, young leaf, mature leaf, old leaf, root, and stem. **(D)** Heatmap of *CsZIPs* gene expression in bud and different leaf positions (first to fourth leaf). The hierarchical clustering tree is shown on the left of each heatmap. Expression values are presented as row-wise normalized TPM data.

The CsZIP proteins ranged from 334 to 375 aa in length, with MW ranging from 35.74 to 39.67 kDa, and GRAVY values of 0.417–0.728; all belonging to hydrophobic proteins. Except for CsZIP1/2/7/12.1, which encoded acidic proteins, the remaining members were alkaline (**Supplementary Table 2**). All CsZIPs possessed a conserved zinc/iron permease domain and contained 5–9 TMDs (**Figure 3A**). Apart from CsZIP6.1, the remaining members shared highly similar three-dimensional protein structures (**Figure 3B**). Subcellular localization prediction indicated that members of Subfamilies III and IV may be localized to chloroplasts, while the others potentially target the cell membrane (**Table 1**). The number of introns in *CsZIPs* varied from 1 to 6; among them, *CsZIP1/3/5/10/12.2* shared identical gene structures, suggesting a close evolutionary relationship (**Supplementary Figure 1A**). A total of 10 conserved motifs were predicted in CsZIP proteins. Motif 2 and Motif 6 were universally conserved across all members, suggesting that these motifs may be essential for the biological functions of CsZIPs (**Supplementary Figures 1B, C**).

**Table 1:** Physicochemical characteristics of *CsZIPs*, *CsNRAMPs*, *CsHMAs*, *CsYSLs*, *CsMTPs*, and *CsCAXs* family in tea plants.

Gene family	Gene name	Gene ID	Subfamily classification	Subcellular localization	Transmembrane domain	Main functions	Evidence type	References
*CsZIPs*	*CsZIP1*	CSS0029626	I	Cell membrane	7	Mediate Zn^2+^ influx from soil into root cells	Experimental verification	Zheng et al., 2022
	*CsZIP2*	CSS0004796	II	Cell membrane	9			
	*CsZIP3*	CSS0022695	I	Cell membrane	7			
	*CsZIP4*	CSS0043874	III	Chloroplast	5			
	*CsZIP5*	CSS0010396	I	Cell membrane	7			
	*CsZIP6.1*	CSS0007950	IV	Chloroplast	8			
	*CsZIP6.2*	CSS0048283	IV	Chloroplast	8			
	*CsZIP7*	CSS0027771	V	Cell membrane	8			
	*CsZIP10*	CSS0036144	V	Cell membrane	8			
	*CsZIP11.1*	CSS0011926	II	Cell membrane	9			
	*CsZIP11.2*	CSS0008965	II	Cell membrane	9			
	*CsZIP12.1*	CSS0045198	I	Cell membrane	7			
	*CsZIP12.2*	CSS0017488	I	Cell membrane	7			
*CsNRAMPs*	*CsNRAMP1*	CSS0031499	I	Cell membrane	12	Divalent cation transporter, involved in Zn^2+^ uptake	Homology prediction	Li et al., 2021; Zhao et al., 2026; Kosuth et al., 2023; Huang et al., 2024, 2024; Meier et al., 2025; Qu and Nakanishi, 2023; Huang et al., 2026
	*CsNRAMP2*	CSS0044220	II	Cell membrane	11			
	*CsNRAMP3.1*	CSS0041547	II	Cell membrane	11			
	*CsNRAMP3.2*	CSS0022297	II	Cell membrane	11			
	*CsNRAMP3.3*	CSS0047249	II	Cell membrane	10			
	*CsNRAMP4*	CSS0021034	II	Cell membrane	11			
	*CsNRAMP5*	CSS0004419	II	Cell membrane	11			
	*CsNRAMP6*	CSS0042984	I	Cell membrane	11			
	*CsNRAMP7*	CSS0046035	III	Cell membrane	12			
	*CsNRAMP8*	CSS0019007	III	Cell membrane	12			
	*CsNRAMP9*	CSS0007121	III	Cell membrane	12			
	*CsNRAMP10*	CSS0046206	III	Cell membrane	12			
*CsHMAs*	*CsHMA1*	CSS0024485	Zn/Co/Cd/Pb	Chloroplast	5	Xylem loading of zinc and root-to-shoot long-distance zinc translocation	Homology prediction	Hussain et al., 2004; Sinclair et al., 2007; Wong and Cobbett, 2009; Hennigar and Kelleher, 2012; Huang et al., 2024
	*CsHMA3*	CSS0005701	Zn/Co/Cd/Pb	Cell membrane	6			
	*CsHMA5.1*	CSS0042325	Cu/Ag	Cell membrane	8			
	*CsHMA5.2*	CSS0024211	Cu/Ag	Cell membrane	8			
	*CsHMA5.3*	CSS0027458	Cu/Ag	Cell membrane	6			
	*CsHMA5.4*	CSS0023767	Cu/Ag	Cell membrane	8			
	*CsHMA6.1*	CSS0044959	Cu/Ag	Cell membrane	8			
	*CsHMA6.2*	CSS0014897	Cu/Ag	Nucleus	0			
	*CsHMA7*	CSS0014840	Cu/Ag	Cell membrane	7			
	*CsHMA8*	CSS0040015	Cu/Ag	Cell membrane	6			
*CsYSLs*	*CsYSL1.1*	CSS0007085	I	Cell membrane	14	Phloem loading of zinc ions and long-distance transport	Homology prediction	DiDonato et al., 2004; Schaaf et al., 2005; Chu et al., 2010; Huang et al., 2024
	*CsYSL1.2*	CSS0025418	I	Cell membrane	13			
	*CsYSL2*	CSS0039421	I	Cell membrane	12			
	*CsYSL3*	CSS0025120	I	Cell membrane	14			
	*CsYSL4*	CSS0026851	II	Cell membrane	14			
	*CsYSL5*	CSS0007763	III	Cell membrane	14			
	*CsYSL6*	CSS0018457	II	Cell membrane	14			
	*CsYSL7*	CSS0025441	III	Cell membrane	14			
	*CsYSL8*	CSS0044292	III	Cell membrane	6			
	*CsYSL9*	CSS0047696	III	Cell membrane	13			
*CsMTPs*	*CsMTP1*	CSS0036317	Zn-CDF	Vacuole	6	Facilitate the sequestration of Zn^2+^ into vacuolar or secretory compartments	Experimental verification	Zhang et al., 2020; Li et al., 2017; Yuan et al., 2017; Zhang et al., 2019; Li et al., 2024b
	*CsMTP2*	CSS0014621	Zn-CDF	Vacuole	6			
	*CsMTP3*	CSS0049997	Zn-CDF	Vacuole	6			
	*CsMTP4*	CSS0028321	Zn-CDF	Vacuole	6			
	*CsMTP5*	CSS0003318	Zn-CDF	Vacuole	5			
	*CsMTP7*	CSS0006652	Zn/Fe-CDF	Vacuole	4			
	*CsMTP8.1*	CSS0043434	Mn-CDF	Vacuole	5			
	*CsMTP8.2*	CSS0042503	Mn-CDF	Vacuole	4			
	*CsMTP8.3*	CSS0033190	Mn-CDF	Vacuole	3			
	*CsMTP9.1*	CSS0044030	Mn-CDF	Vacuole	5			
	*CsMTP9.2*	CSS0037602	Mn-CDF	Vacuole	2			
	*CsMTP10*	CSS0023067	Mn-CDF	Vacuole and Cell membrane	5			
	*CsMTP11.1*	CSS0026766	Mn-CDF	Vacuole	5			
	*CsMTP11.2*	CSS0018656	Mn-CDF	Vacuole and Cell membrane	4			
*CsCAXs*	*CsCAX1*	CSS0036754	I	Vacuole	10	Sequester excess Zn^2+^ from the cytosol into vacuolar compartments, thereby preserving cytosolic zinc homeostasis	Homology prediction	Koren’kov et al., 2006; Cheng et al., 2005; Huang et al., 2024
	*CsCAX2*	CSS0007722	I	Vacuole	10			
	*CsCAX3*	CSS0006886	I	Vacuole	10			
	*CsCAX4*	CSS0034222	I	Vacuole	10			
	*CsCAX5*	CSS0000893	II	Vacuole	11			
	*CsCAX6*	CSS0026495	III	Cell membrane and Golgi apparatus	10			
	*CsCAX7*	CSS0007913	III	Cell membrane and Golgi apparatus	13			
	*CsCAX8*	CSS0002251	V	Cell membrane and Chloroplast	13			
	*CsCAX9.1*	CSS0006324	IV	Cell membrane	12			
	*CsCAX9.2*	CSS0015661	IV	Cell membrane	6			
	*CsCAX10*	CSS0049964	V	Cell membrane and Chloroplast	11			
	*CsCAX11*	CSS0031507	V	Chloroplast	13			
	*CsCAX12*	CSS0018394	V	Cell membrane, Chloroplast and Mitochondrion	12			
	*CsCAX13*	CSS0020087	V	Chloroplast	7			
	*CsCAX14*	CSS0024397	V	Chloroplast	7			

*CsZIPs* exhibited distinct tissue-specific, developmentally regulated, and stress-induced expression patterns, and are closely associated with catechin and amino acid accumulation and tea quality formation (Zheng et al., 2022). *CsZIP1/3/12.2*, *CsZIP2/4/10/11.1*, *CsZIP6.1/6.2/11.2*, and *CsZIP5/12.1* were predominantly expressed in stems, roots, fruits, and leaves, respectively (**Figure 3C**). During leaf development, the overall expression level of *CsZIPs* in buds was lower than in mature leaves, and *CsZIP12.1/12.2* were highly expressed in the second leaf (**Figure 3D**). The promoter regions of *CsZIPs* were enriched in numerous *cis*-acting elements, such as MYC, MYB, and Box4. *CsZIP6.1* contained 10 abscisic acid (ABA)-responsive elements (ABRE), and *CsZIP3* contained 7 salicylic acid (SA)-responsive elements (TCA-element), indicating that they may participate in ABA and SA signaling pathways, respectively (**Supplementary Figure 2**).

In summary, 13 *CsZIP* genes were identified in the tea plant genome, primarily participating in Zn^2+^ uptake and transport. Eight members have been functionally validated by yeast complementation, and *CsZIP4* was further confirmed to mediate Zn-nitrogen coordinated uptake in planta (Zheng et al., 2022; Xu et al., 2024). These CsZIPs were divided into five subfamilies, with one intraspecific collinear gene pair subjected to purifying selection, and their evolutionary pattern was more similar to that of woody plants. The majority of CsZIPs were hydrophobic alkaline proteins containing a typical zinc/iron permease domain and 5–9 TMDs, mainly localized to the cell membrane, with some members targeted to chloroplasts. *CsZIPs* displayed tissue and developmental stage-specific expression, and their promoters were enriched in MYC and MYB *cis*-elements. Notably, *CsZIP6.1* was rich in ABRE and *CsZIP3* contained abundant TCA-elements, implying their involvement in ABA- and SA-mediated regulatory pathways.

### 3.2 The CsNRAMP protein family in tea plants

**Figure 4:**
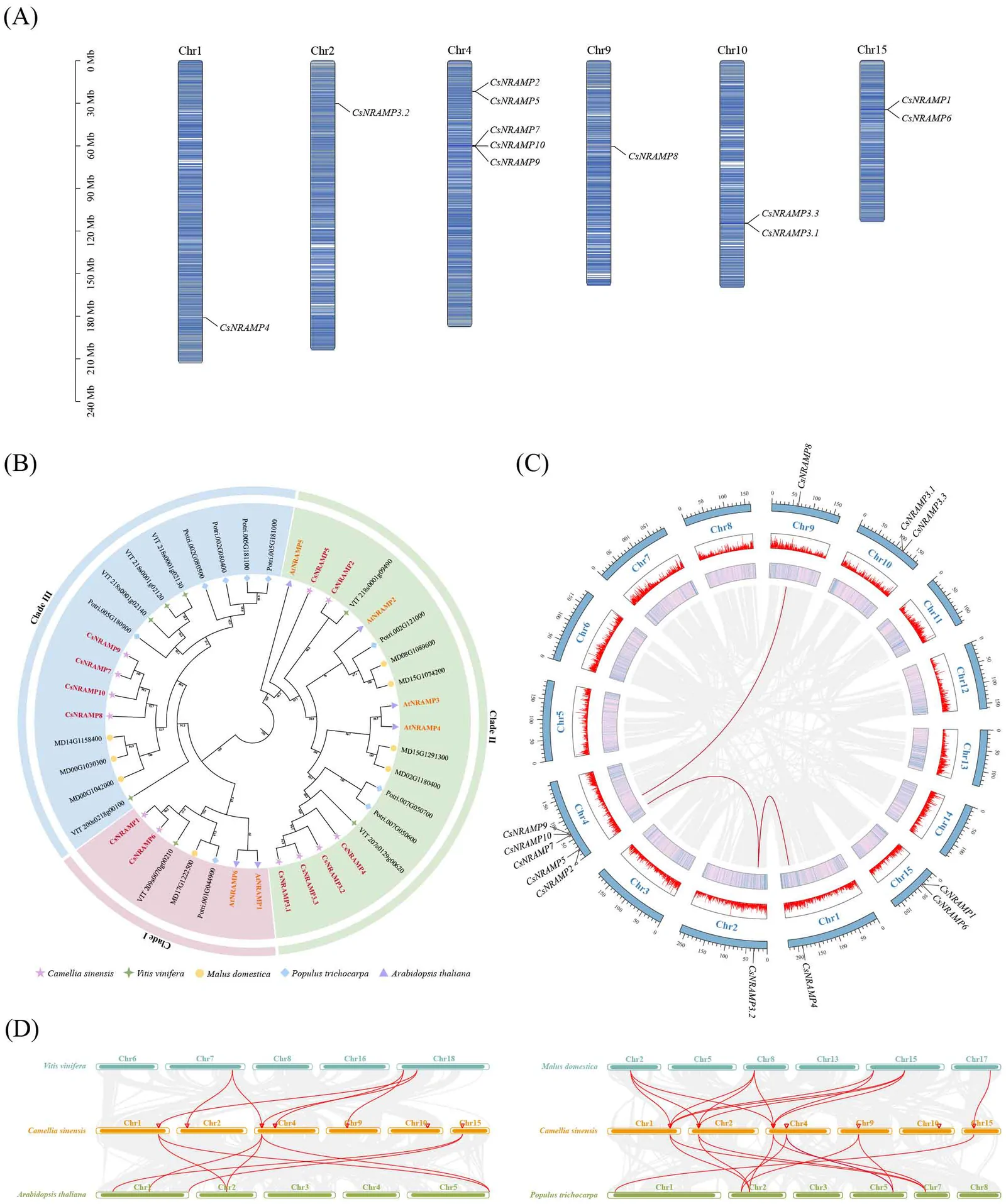
Chromosome distribution, phylogenetic relationship, and synteny analysis of *CsNRAMP* gene family. **(A)** Chromosomal localization of *CsNRAMPs* in tea plant. **(B)** Phylogenetic relationship of NRAMP family proteins from tea plants and other plants. The phylogenetic tree was constructed using the neighbor-joining (NJ) method in MEGA11 with 1000 bootstrap replicates. **(C)** Intraspecies collinearity analysis of *CsNRAMPs* in tea plants. Gray lines represent all collinear blocks within the tea genome, and red lines highlight duplicated *CsNRAMPs* gene pairs. **(D)** Interspecies collinearity analysis among tea plant, *Vitis vinifera*, *Arabidopsis*, *Populus trichocarpa*, and *Malus domestica*. Gray lines represent syntenic blocks across genomes, and red lines denote orthologous *CsNRAMP* relationships.

Natural resistance-associated macrophage protein (NRAMP) is a class of conserved divalent metal transporters that play essential roles in maintaining plant micronutrient homeostasis (Nevo and Nelson, 2006). A total of 12 *CsNRAMP* genes were identified in the tea plant genome and were unevenly distributed across six chromosomes (**Figure 4A**). Phylogenetic analysis classified these CsNRAMPs into three subfamilies. Subfamilies I and II potentially shared closer genetic relationships with *Arabidopsis* NRAMP members, while Subfamily III may form a tea-specific evolutionary branch with no homologous genes in *Arabidopsis* (**Figure 4B**). Intragenomic collinearity analysis identified three collinear gene pairs, all with Ka/Ks values <1, indicating that they may have undergone purifying selection (**Figure 4C**, **Supplementary Table 1**). The number of collinear gene pairs between tea plants and apple/poplar was greater than that between tea plants and *Arabidopsis*/grape, suggesting that tea plants potentially share a closer evolutionary affinity with apple and poplar (**Figure 4D**).

**Figure 5:**
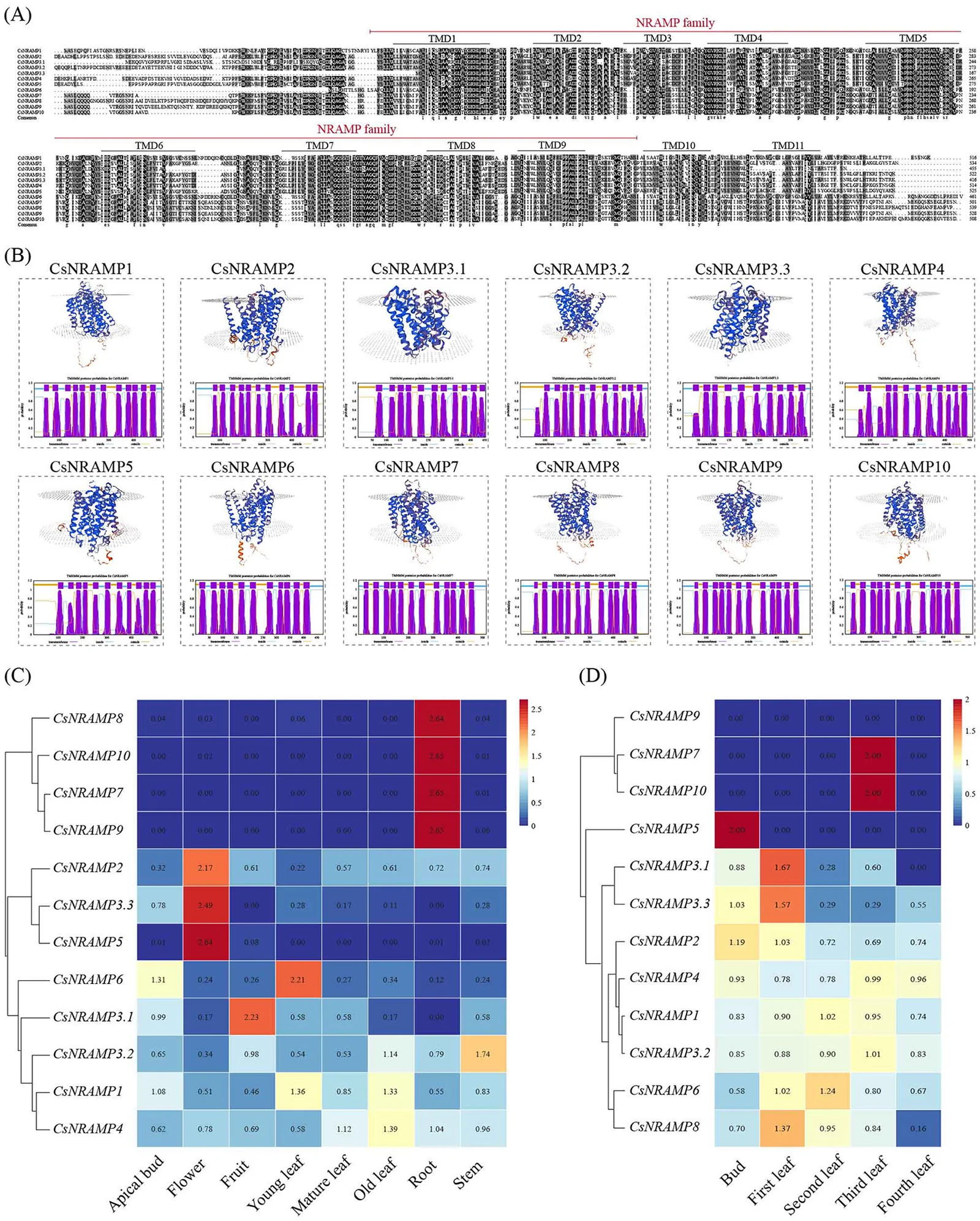
Multiple sequence alignment, three-dimensional structure modeling, transmembrane domain prediction, and tissue-specific expression profiling of *CsNRAMPs* in tea plant. **(A)** Multiple sequence alignment of CsNRAMP proteins. Black bars above the sequences indicate TMDs, while red bars denote the NRAMP family signature domain. Alignment shading scheme: black, ≥75% conservation; dark gray, ≥50% conservation; light gray, ≥33% conservation; dots, gaps. **(B)** Predicted transmembrane domains and three-dimensional structures of CsNRAMP proteins. The disk-shaped structure composed of gray dots in the three-dimensional protein models represents the membrane. In the transmembrane domain probability plots, purple peaks indicate transmembrane regions, the blue line corresponds to the intracellular compartment, and the yellow line corresponds to the extracellular compartment. **(C)** Heatmap showing *CsNRAMPs* gene expression across eight tissues: apical bud, flower, fruit, young leaf, mature leaf, old leaf, root, and stem. **(D)** Heatmap showing *CsNRAMPs* gene expression in apical bud and leaves at different developmental stages (first to fourth leaf). Hierarchical clustering trees are displayed to the left of each heatmap. Expression values are presented as row-wise normalized TPM data..

The length of CsNRAMP proteins ranged from 416 to 555 aa, with MWs ranging from 45.7 to 60.64 kDa. Proteins in Subfamilies I and III were alkaline, whereas most members of Subfamily II were acidic (**Supplementary Table 2**). All CsNRAMPs were hydrophobic membrane proteins containing a typical conserved NRAMP domain and 10–12 TMDs, with highly similar tertiary protein structures (**Table 1**, **Figures 5A, B**). Gene structure varied considerably among subfamilies: Subfamily I contained 10–13 exons; Subfamily II exhibited a conserved gene structure with predominantly four exons; and all members of Subfamily III consisted of 13 exons and 12 introns (**Supplementary Figure 3A**). A total of 10 conserved motifs were identified in tea CsNRAMP proteins. Motifs 2, 3, 4, 5, 7, and 8 were shared by all family members, suggesting that they constitute indispensable core motifs of the NRAMP structural domain (**Supplementary Figures 3B, C**).

*CsNRAMPs* exhibited distinct tissue specificity and spatiotemporal expression patterns. In different tea plant tissues, Subfamily III genes showed root-specific expression; *CsNRAMP2/3.3/5* were significantly highly expressed in flowers, while the other members displayed no obvious tissue preference (**Figure 5C**). During leaf development, *CsNRAMP5*, *CsNRAMP3.1/3.3*, and *CsNRAMP7/9* were predominantly expressed in buds, the first leaf, and the third leaf, respectively, whereas the expression of other members showed no obvious developmental variation (**Figure 5D**). The promoters of all *CsNRAMPs* contained MYC and MYB response elements and were enriched in *cis*-acting elements, such as ARE, STRE, and Box4. Among them, *CsNRAMP7/9* contained abundant ABRE elements, implying that they may potentially participate in ABA signaling regulation (**Supplementary Figure 4**).

In summary, 12 CsNRAMP genes were identified in tea plants and classified into three subfamilies. Subfamilies I and II were closely related to *Arabidopsis* homologs, while Subfamily III was tea-specific and evolutionarily closer to woody plants such as apple and poplar. CsNRAMPs showed subfamily-specific differences in physicochemical properties and were all structurally conserved hydrophobic membrane proteins with marked differences in gene structure among subfamilies. The *CsNRAMPs* displayed prominent tissue and spatiotemporal expression patterns: Subfamily III was specifically expressed in roots, and *CsNRAMP2/3.3/5* were highly expressed in flowers. Their promoters were enriched in MYC/MYB *cis*-elements, and *CsNRAMP7/9* contained abundant ABRE elements, suggesting a role in ABA-mediated signaling pathways.

### 3.3 Xylem loading and long-distance transport: CsHMAs

**Figure 6:**
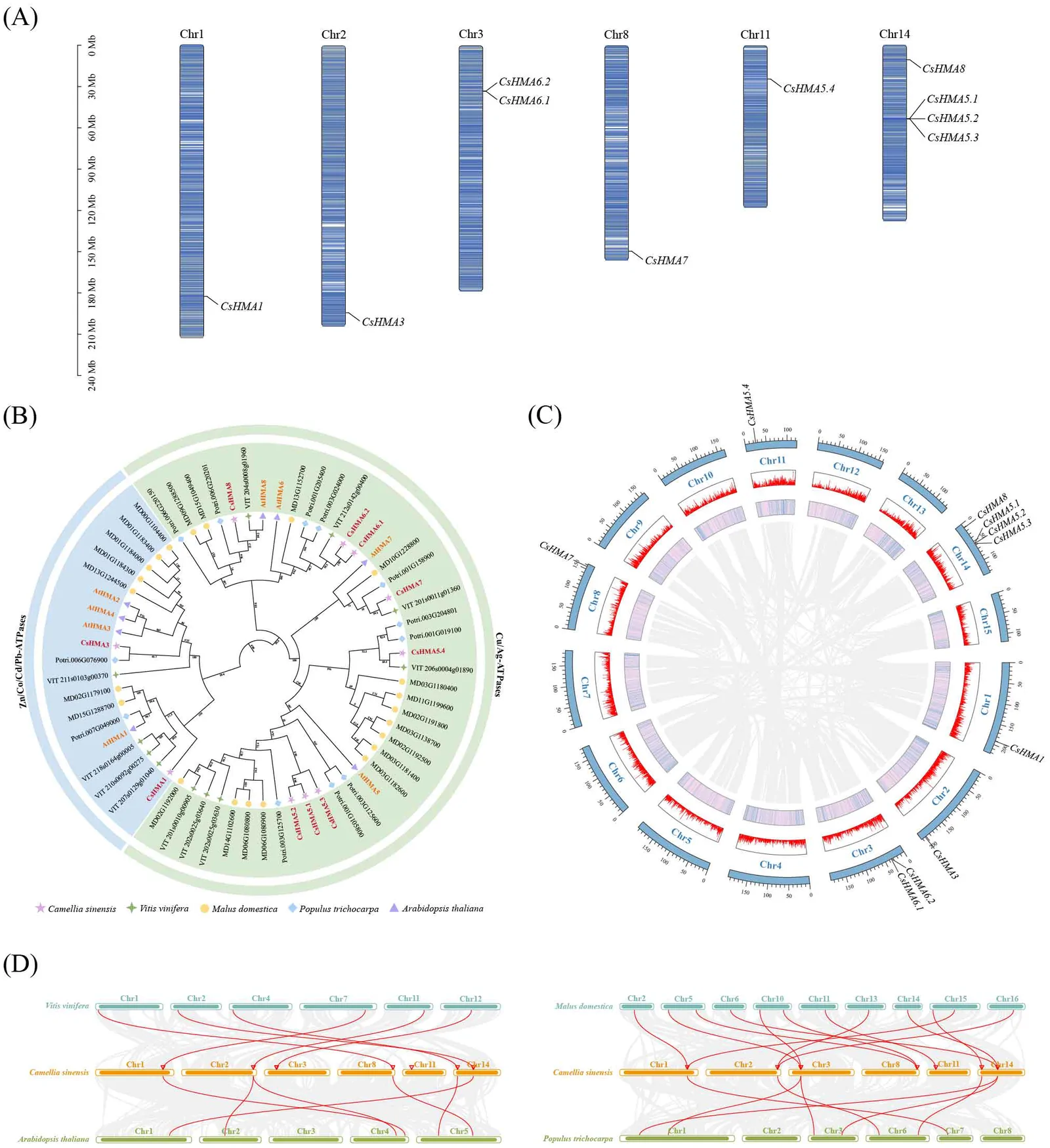
Chromosome distribution, phylogenetic relationship, and synteny analysis of *CsHMA* gene family. **(A)** Chromosomal localization of *CsHMAs* in the tea plant genome. **(B)** Phylogenetic relationship of HMA family proteins from tea plants and other plants. The phylogenetic tree was constructed using the neighbor-joining (NJ) method in MEGA11 with 1000 bootstrap replicates. **(C)** Intraspecies collinearity analysis of *CsHMAs* in tea plants. Gray lines represent all collinear blocks within the tea genome, and red lines highlight duplicated *CsHMAs* gene pairs. **(D)** Interspecies collinearity analysis among tea plant, *Vitis vinifera*, *Arabidopsis*, *Populus trichocarpa*, and *Malus domestica*. Gray lines represent syntenic blocks across genomes, and red lines denote orthologous *CsHMA* relationships.

Heavy metal ATPases (HMA/P1B-ATPases) hydrolyze ATP to drive transmembrane transport of heavy metal cations, and represent the only group within the P-type ATPase superfamily involved in metal ion homeostasis (Argüello, 2003). A total of 10 *CsHMA* genes were identified in the tea plant genome and were unevenly distributed on chromosomes 1, 2, 3, 8, 11, and 14 (**Figure 6A**). The *Arabidopsis* HMA family is divided into two major clades: the Cu/Ag-ATPase clade, responsible for copper and silver transport, and the Zn/Co/Cd/Pb-ATPase clade, which mediates the translocation of zinc, cobalt, cadmium, and lead (Williams and Mills, 2005). Phylogenetic analysis showed that CsHMA1 and CsHMA3 belonged to the Zn/Co/Cd/Pb-ATPase subfamily; while CsHMA5.1-CsHMA5.4, CsHMA6.1-CsHMA6.2, CsHMA7, and CsHMA8 clustered within the Cu/Ag-ATPase subfamily (**Figure 6B**). No duplicated segments of *CsHMAs* were detected in the tea plant genome, suggesting that whole-genome or segmental duplication did not contribute to the expansion of this family (**Figure 6C**). Tea plants shared the most HMA collinear gene pairs with apple (12 pairs), whereas only 6–7 collinear pairs were detected with poplar, grape, and *Arabidopsis*. These results may suggest that tea plants share a closer evolutionary relationship with apple than with the other species analyzed (**Figure 6D**).

**Figure 7:**
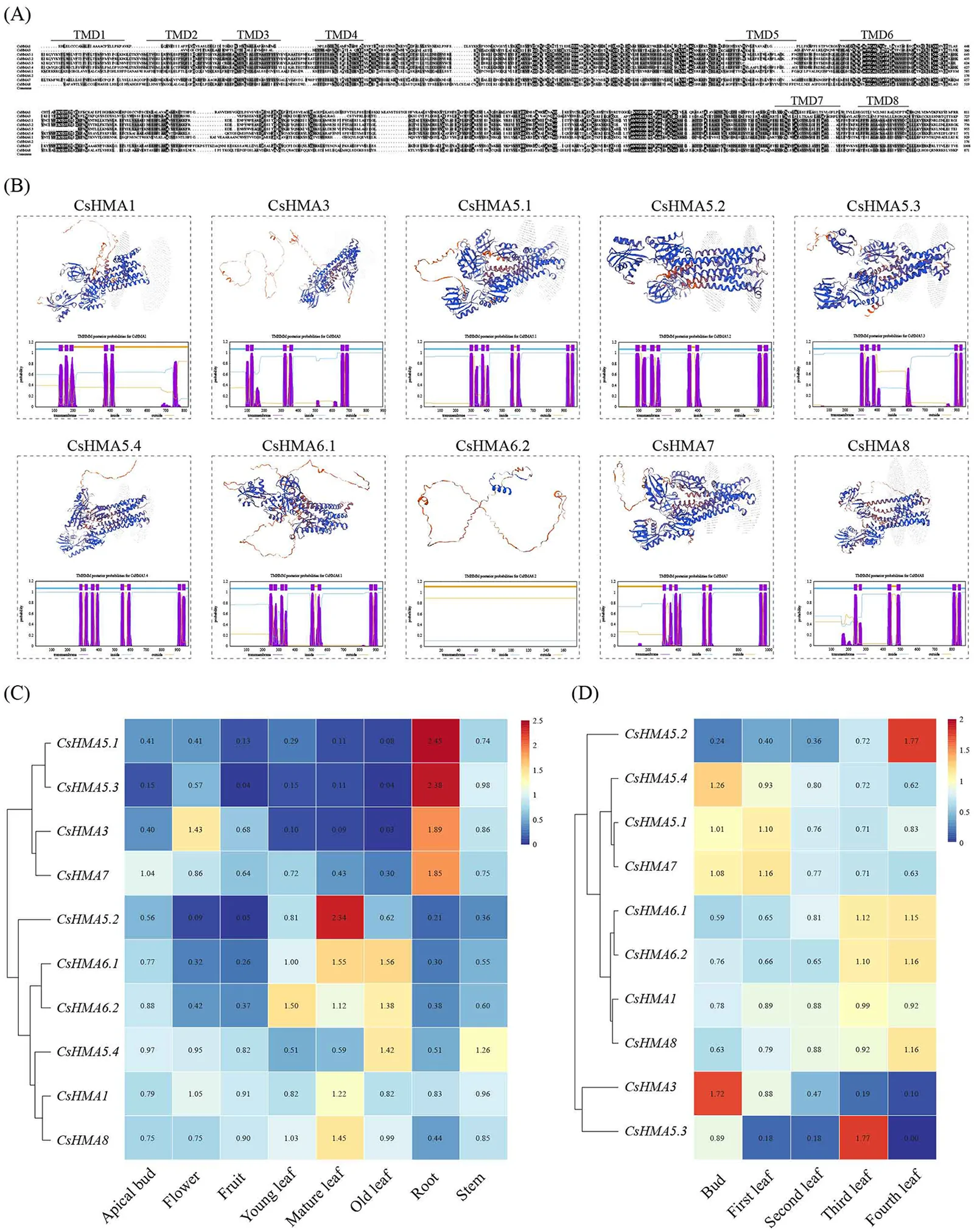
Multiple sequence alignment, three-dimensional structure modeling, transmembrane domain prediction, and tissue-specific expression profiling of CsHMAs in tea plant. **(A)** Multiple sequence alignment analysis of CsHMA proteins in tea plants. The black bars above the sequences denote the TMDs. Alignment coloring: black, ≥75% conservation; dark gray, ≥50% conservation; light gray, ≥33% conservation; dots, gaps. **(B)** Predicted transmembrane domains and three-dimensional structures of CsHMA proteins. In the structural models, the disk-shaped structure composed of gray dots represents the lipid bilayer membrane. In the transmembrane probability plots, purple peaks indicate transmembrane regions, the blue line corresponds to the intracellular compartment, and the yellow line corresponds to the extracellular compartment. **(C)** Heatmap of *CsHMAs* gene expression in apical bud, flower, fruit, young leaf, mature leaf, old leaf, root, and stem. **(D)** Heatmap of *CsHMAs* gene expression in bud and different leaf positions (first to fourth leaf). The hierarchical clustering tree is shown on the left of each heatmap. Expression values are presented as row-wise normalized TPM data..

HMA proteins typically contain 6–8 TMDs, along with metal-binding sites and regulatory domains that determine substrate specificity (Smith et al., 2014). The CsHMA proteins ranged from 176 to 1008 aa in length, with MWs of 18.00 to 107.83 kDa. All CsHMAs had positive GRAVY values and were classified as hydrophobic proteins (**Supplementary Table 2**). Members of the Zn/Co/Cd/Pb-ATPase subfamily were alkaline proteins, and except for CsHMA6.1/6.2, the majority of members of the Cu/Ag-ATPase subfamily were acidic proteins, which may reflect marked physicochemical differences between the two subfamilies that facilitate adaptation to distinct microenvironments (**Supplementary Table 2**). With the exception of CsHMA6.2, the other nine CsHMAs contained 5–8 TMDs. The majority of CsHMAs may localize to the cell membrane; CsHMA1 putatively targeted chloroplasts and CsHMA6.2 potentially resided in the nucleus, implying this family may exert versatile functions in metal transport across both the cell membrane and organellar membranes (**Table 1**). CsHMAs exhibited highly conserved amino acid sequences, and their three-dimensional structures generally contained 5–8 transmembrane helices, consistent with the typical structural features of P1B-type ATPases (**Figures 7A, B**). *CsHMAs* displayed substantial variation in gene structure, with 1–16 introns and 2–17 exons (**Supplementary Figure 5A**). MEME analysis identified 10 conserved motifs. CsHMA6.2 lacked conserved motifs, suggesting remarkable functional divergence; CsHMA5.1–CsHMA5.4 contained all 10 motifs with high sequence conservation, and each phylogenetic clade displayed a subfamily-specific motif distribution pattern (**Supplementary Figures 5B, C**).

In different tea plant tissues, *CsHMA1/5.2/5.4/6.1/6.2/8* were predominantly expressed in leaves, while *CsHMA3/5.1/5.3/7* were highly expressed in roots and stems, suggesting that they may participate in root-to-shoot heavy metal translocation (**Figure 7C**). During leaf development, *CsHMA3*, *CsHMA5.2*, and *CsHMA5.3* were preferentially expressed in buds, the fourth leaf, and the third leaf, respectively. With leaf maturation, the expression of *CsHMA5.1/5.3/5.4/7* gradually declined, whereas the transcript levels of *CsHMA1/5.2/6.1/6.2/8* increased progressively (**Figure 7D**). The promoter regions of *CsHMAs* were enriched in multiple functional *cis*-elements, including ARE, MYB, MYC, STRE, BOX4, GT1-motif, ERE, and ABRE, which may potentially mediate stress response, light signaling, and hormonal regulation in tea plants. Notably, the *CsHMA8* promoter contained 12 ethylene-responsive elements (ERE), suggesting its potential participation in ethylene signaling pathways (**Supplementary Figure 6**).

In summary, 10 *CsHMA* genes were identified in tea plants and classified into two subfamilies: Zn/Co/Cd/Pb-ATPases and Cu/Ag-ATPases. The CsHMA family had not undergone expansion through gene duplication and showed the closest evolutionary relationship with apple. The majority of CsHMAs were hydrophobic proteins mainly localized to the cell membrane; the Zn subfamily consisted of alkaline proteins, while the Cu subfamily was predominantly acidic. Gene structure varied significantly among members, and CsHMA6.2 lacked conserved motifs, suggesting functional divergence. *CsHMAs* exhibited distinct tissue- and development-specific expression patterns and participated in root-to-shoot heavy metal transport. Their promoters were enriched in diverse stress- and hormone-responsive elements, and *CsHMA8* was rich in ERE motifs, implying its potential role in ethylene signaling regulation.

### 3.4 Phloem loading and grain/leaf enrichment: CsYSLs

**Figure 8:**
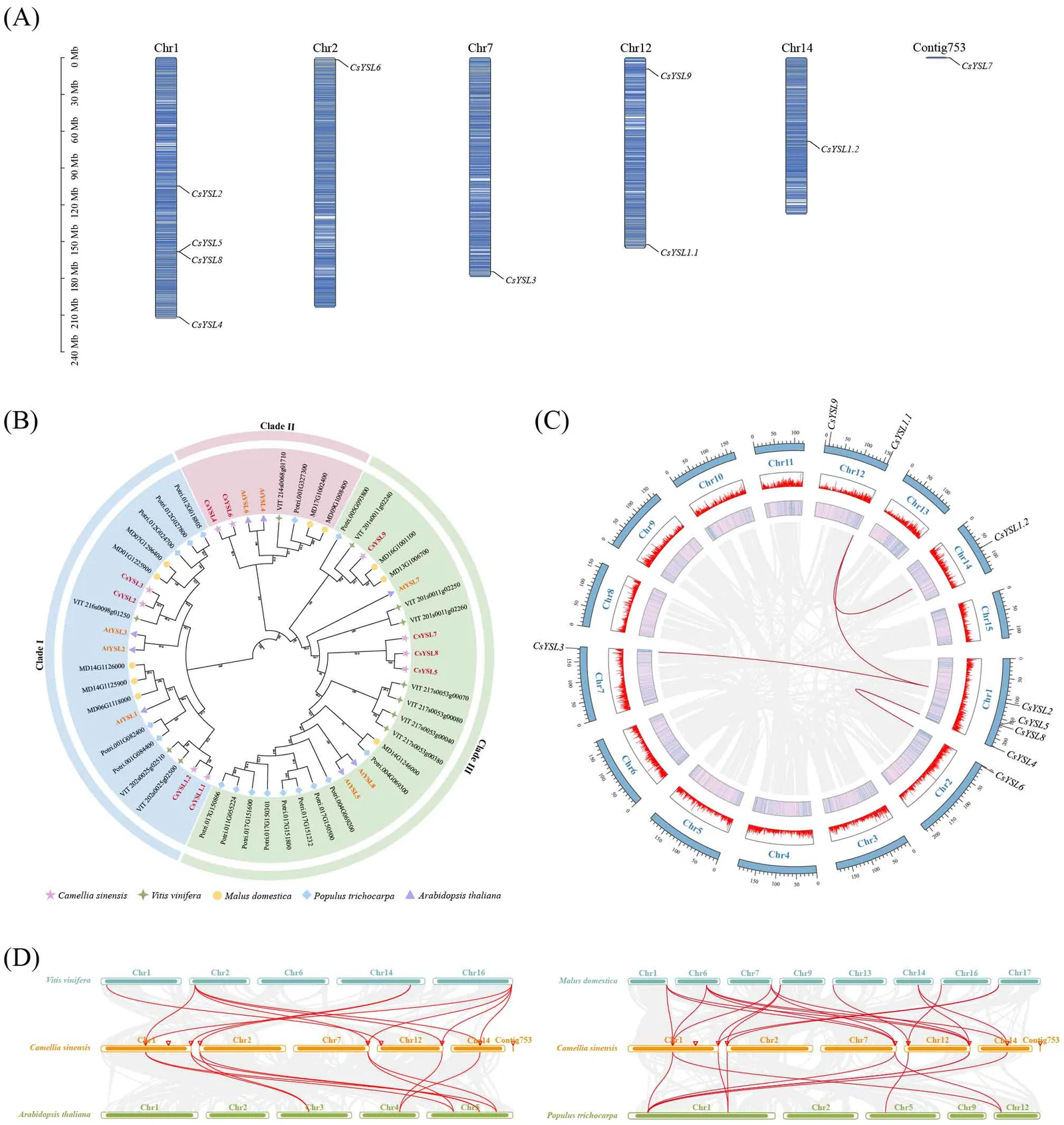
Chromosome distribution, phylogenetic relationship, and synteny analysis of *CsYSL* gene family. **(A)** Chromosomal localization of *CsYSLs* in tea plants. **(B)** Phylogenetic relationship of YSL family proteins from tea plants and other plant species. The phylogenetic tree was constructed using the neighbor-joining (NJ) method in MEGA11 with 1000 bootstrap replicates. **(C)** Intraspecies collinearity analysis of *CsYSLs* in tea plants. Gray lines represent all collinear blocks within the tea genome, and red lines highlight duplicated *CsYSL* gene pairs. **(D)** Interspecies collinearity analysis among tea plant, *Vitis vinifera*, *Arabidopsis*, *Populus trichocarpa*, and *Malus domestica*. Gray lines represent syntenic blocks across genomes, and red lines denote orthologous *CsYSL* relationships.

Yellow stripe-like (YSL) proteins belong to the oligopeptide transporter (OPT) superfamily and function as heavy metal transporters. A total of 10 *CsYSL* genes were identified in the tea plant genome. Among them, *CsYSL7* was located on scaffold contig753, while the remaining members were unevenly distributed on chromosomes 1, 2, 7, 12, and 14 (**Figure 8A**). Plant YSLs are generally classified into two groups according to substrate specificity: one group transports Fe (III)-phytosiderophore complexes, and the other mediates the translocation of metal-NA complexes (Curie et al., 2009). Phylogenetic analysis divided CsYSLs into three subfamilies (I, II, and III). Notably, CsYSL4/6 clustered into a unique clade with six YSL members from other plant species, implying they may perform distinctive physiological functions (**Figure 8B**). Four collinear gene pairs were identified within the tea genome, all with Ka/Ks values <1, indicating that these gene pairs may have undergone purifying selection during evolution (**Figure 8C**; **Supplementary Table 1**). Interspecific collinearity analysis showed that, except for *CsYSL5/7/8*, all other *CsYSLs* had collinear orthologs in apple, poplar, grape, and *Arabidopsis*. This suggests that *CsYSL5/7/8* may have undergone species-specific divergence and may form tea-specific evolutionary branches (**Figure 8D**).

**Figure 9:**
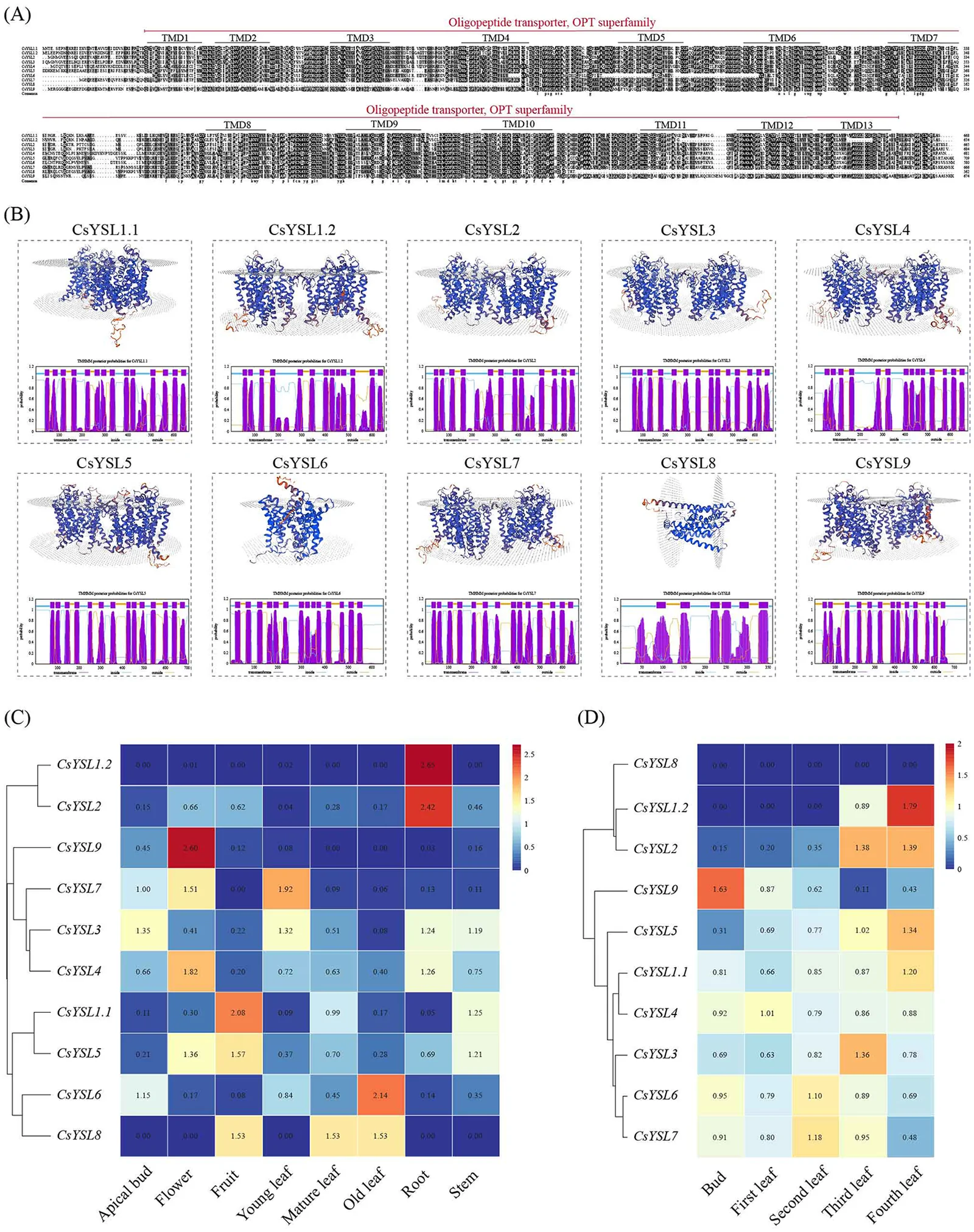
Multiple sequence alignment, three-dimensional structure modeling, transmembrane domain prediction, and tissue-specific expression profiling of CsYSLs in tea plants. **(A)** Multiple sequence alignment analysis of CsYSL proteins in tea plant. The black bars above the sequences denote the TMDs. The red bars above the sequences denote the oligopeptide transporter (OPT) superfamily domain. Alignment coloring: black, ≥75% conservation; dark gray, ≥50% conservation; light gray, ≥33% conservation; dots, gaps. **(B)** Predicted transmembrane domains and three-dimensional structures of CsYSL proteins. The disk-shaped structure composed of gray dots in the three-dimensional protein models represents the membrane. In the transmembrane domain probability plots, purple peaks indicate transmembrane regions, the blue line corresponds to the intracellular compartment, and the yellow line corresponds to the extracellular compartment. **(C)** Heatmap of *CsYSLs* gene expression in apical bud, flower, fruit, young leaf, mature leaf, old leaf, root, and stem. **(D)** Heatmap of *CsYSL* gene expression in bud and different leaf positions (first to fourth leaf). The hierarchical clustering tree is shown on the left of each heatmap. Expression values are presented as row-wise normalized TPM data..

The CsYSL proteins ranged from 362 to 766 aa in length, with MWs of 40.45–83.46 kDa. All CsYSLs had positive GRAVY values and were hydrophobic. Subfamily II members were acidic proteins, whereas the isoelectric points of the remaining members ranged from 8.62 to 9.12, indicating that they are alkaline proteins. This may indicate that Subfamily II potentially underwent functional divergence to adapt to distinct microenvironments (**Supplementary Table 2**). As heavy metal transporters of the OPT superfamily, all CsYSLs contained a conserved OPT domain (**Figure 9A**). YSL proteins are predominantly localized to the cell membrane, with a few targeted to organellar membranes (Curie et al., 2009). They mediate the transmembrane transport of metal-NA chelates and are essential for long-distance metal translocation in plants (Koh et al., 2002; Murata et al., 2006). Subcellular localization prediction revealed that all 10 tea CsYSLs were localized to the cell membrane; aside from CsYSL8, they contained 12–14 TMDs, consistent with their hydrophobic properties (**Table 1**). Three-dimensional protein structure prediction further supported the abundant transmembrane topological features of CsYSLs (**Figure 9B**). *CsYSLs* contained 3–8 introns and 4–9 exons. The intron length of *CsYSL1.2* was markedly longer than those of the other members, suggesting that this long intron may contain specific regulatory elements and potentially drive functional divergence by modulating gene expression and alternative splicing (**Supplementary Figure 7A**). The majority of CsYSLs shared highly conserved motif distribution patterns, while CsYSL6 and CsYSL8 exhibited partial motif loss, implying that motif variation during evolution may contribute to functional differentiation within the family (**Supplementary Figures 7B, C**).

*CsYSLs* exhibited distinct tissue-specific expression patterns. *CsYSL1.2/2* were specifically and highly expressed in roots, suggesting they may participate in root metal ion uptake. *CsYSL9* was predominantly expressed in flowers, *CsYSL1.1/5* were highly expressed in fruits, and *CsYSL6/8* showed relatively high transcript abundance in leaves (**Figure 9C**). During leaf development, *CsYSL8* was barely expressed; the expression of *CsYSL1.2/2/5* gradually increased with leaf maturation, whereas *CsYSL9* displayed the opposite trend. The expression levels of other members showed minor developmental changes (**Figure 9D**). The promoter regions of *CsYSLs* were enriched in *cis*-elements, such as ARE, MYC, MYB, W-box, and Box4. *CsYSL9* contained 30 light-responsive elements, far more than the 10–20 elements present in other members, and uniquely contained 11 Sp1 elements that were absent in all other *CsYSLs*. This suggests that Sp1 elements may be closely associated with the biological function and flower-preferential expression pattern of *CsYSL9* (**Supplementary Figure 8**).

In summary, 10 *CsYSL* genes were identified in the tea plant genome, encoding heavy metal transporters of the OPT superfamily. These CsYSLs were classified into three subfamilies; CsYSL4/6 formed a unique clade, and CsYSL5/7/8 represented tea-specific branches that have undergone purifying selection. All CsYSLs were membrane-localized hydrophobic proteins containing a conserved OPT domain and 12–14 TMDs. Gene structural variation was observed among members, and the long intron of *CsYSL1.2* may regulate gene expression and alternative splicing. The *CsYSL* family showed distinct tissue and leaf developmental spatiotemporal expression patterns. Their promoters were enriched in stress- and light-responsive *cis*-elements, and the unique Sp1 elements in *CsYSL9* may determine its flower-preferential expression.

### 3.5 The CsMTP protein family in tea plants

**Figure 10:**
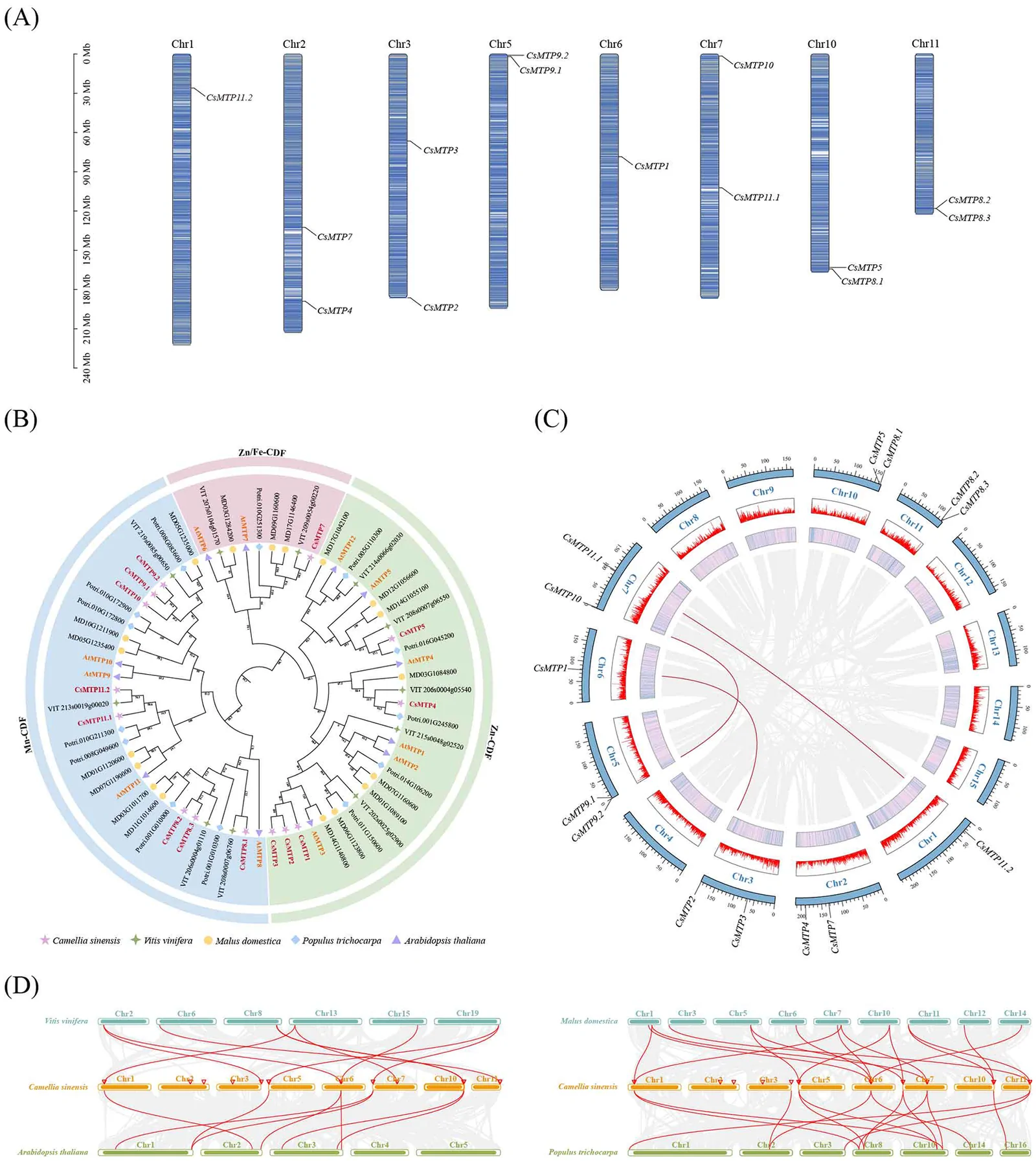
Chromosome distribution, phylogenetic relationship, and synteny analysis of *CsMTP* gene family. **(A)** Chromosomal localization of *CsMTPs* in tea plants. **(B)** Phylogenetic relationship of MTP family proteins from tea plants and other plants. The phylogenetic tree was constructed using the neighbor-joining (NJ) method in MEGA11 with 1000 bootstrap replicates. **(C)** Intraspecies collinearity analysis of *CsMTPs* in tea plant. Gray lines represent all collinear blocks within the tea genome, and red lines highlight duplicated *CsMTPs* gene pairs. **(D)** Interspecies collinearity analysis among tea plant, *Vitis vinifera*, *Arabidopsis*, *Populus trichocarpa*, and *Malus domestica*. Gray lines represent syntenic blocks across genomes, and red lines denote orthologous *CsMTP* relationships.

The cation diffusion facilitator (CDF)/metal tolerance protein (MTP) family comprises essential transporters that regulate Zn^2+^ distribution and homeostasis in plants (Gupta et al., 2016). A total of 14 *CsMTP* genes were identified in the tea plant genome and were unevenly distributed on eight chromosomes (**Figure 10A**). Based on metal substrate specificity, plant MTPs can be divided into three subfamilies: Zn-CDF, Mn-CDF, and Zn/Fe-CDF (Montanini et al., 2007; Zhang et al., 2020). Phylogenetic analysis showed that CsMTP1–5 belonged to the Zn-CDF subfamily; CsMTP8.1-8.3, CsMTP9.1, CsMTP10, and CsMTP11.1/11.2 were clustered into the Mn-CDF subfamily; while CsMTP7 was the sole member of the Zn/Fe-CDF subfamily, suggesting it may possess unique physiological functions (**Figure 10B**). Intragenomic collinearity analysis identified three collinear gene pairs, all with Ka/Ks values <1, indicating that these genes may have undergone purifying selection during evolution (**Figure 10C**; **Supplementary Table 1**). Interspecific collinearity revealed that the number of collinear pairs between tea plant and apple (16 pairs) and poplar (14 pairs) was markedly higher than that with *Arabidopsis* (8 pairs) and grape (9 pairs), implying that tea plants may share a closer evolutionary affinity with apple and poplar (**Figure 10D**).

**Figure 11:**
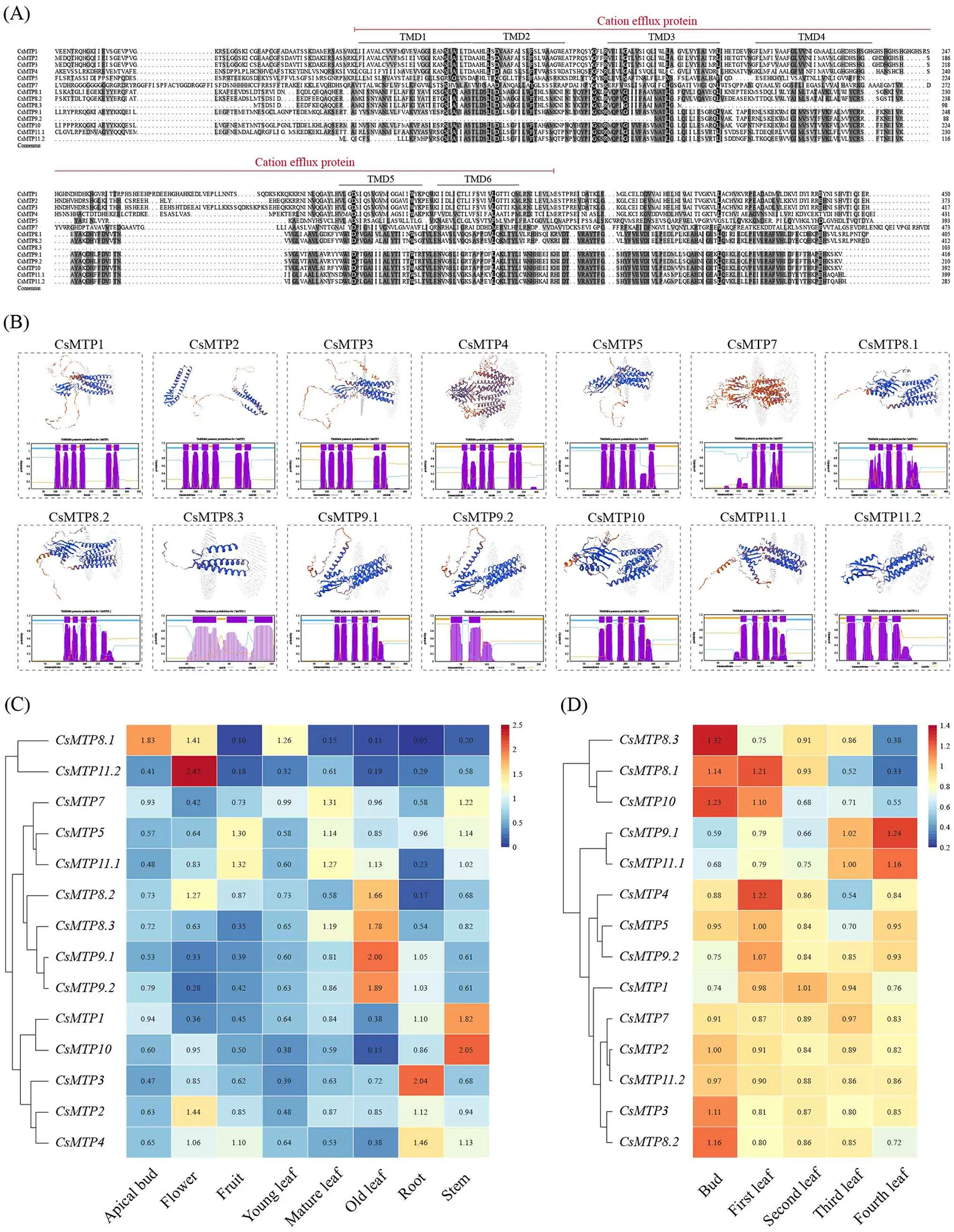
Multiple sequence alignment, three-dimensional structure modeling, transmembrane domain prediction, and tissue-specific expression profiling of CsMTPs in tea plant. **(A)** Multiple sequence alignment analysis of CsMTP proteins in tea plants. The black bars above the sequences denote the TMDs. The red bars above the sequences denote the cation efflux protein domain. Alignment coloring: black, ≥75% conservation; dark gray, ≥50% conservation; light gray, ≥33% conservation; dots, gaps. **(B)** Predicted transmembrane domains and three-dimensional structures of CsMTP proteins. The disk-shaped structure composed of gray dots in the three-dimensional protein models represents the membrane. In the transmembrane domain probability plots, purple peaks indicate transmembrane regions, the blue line corresponds to the intracellular compartment, and the yellow line corresponds to the extracellular compartment. **(C)** Heatmap of *CsMTPs* gene expression in apical bud, flower, fruit, young leaf, mature leaf, old leaf, root, and stem. **(D)** Heatmap of *CsMTPs* gene expression in bud and different leaf positions (first to fourth leaf). The hierarchical clustering tree is shown on the left of each heatmap. Expression values are presented as row-wise normalized TPM data..

The CsMTP proteins ranged from 103 to 483 aa in length, with MWs of 11.4–52.81 kDa. Among them, CsMTP8.2/9.1/10 were hydrophilic, while the rest were hydrophobic. The majority of CsMTPs were acidic proteins, and only CsMTP7 of the Zn/Fe-CDF subfamily was alkaline, which may reflect marked physicochemical differences among subfamilies (**Supplementary Table 2**). Subcellular localization prediction showed that all CsMTPs were targeted to the vacuole, whereas CsMTP10/11.2 were additionally localized to the cell membrane, consistent with the cell membrane localization of CsMTP8.2 verified by heterologous expression in tobacco (Zhang et al., 2020). Except for CsMTP5/7, all other members contained a highly conserved cation efflux domain (**Figure 11A**). Most CsMTP proteins possessed 4–6 TMDs, whereas CsMTP8.3 and CsMTP9.2 contained only 3 and 2 TMDs, respectively. The three-dimensional structures of Mn-CDF and Zn-CDF subfamily proteins were highly conserved; CsMTP4 may exhibit a specific structure, and CsMTP7 potentially showed distinct structural divergence from the other two subfamilies (**Figure 11B**). The exon-intron organization of *CsMTP7* differed greatly from that of the other members (**Supplementary Figure 9A**), and CsMTP5/7 lacked conserved protein motifs, consistent with the domain alignment results (**Supplementary Figures 9B, C**).

Previous studies have confirmed that *CsMTP8* is highly expressed in leaf tissues (Li et al., 2017). Among eight tea plant tissues, *CsMTP1/2/3/4/10* were predominantly expressed in roots and stems, *CsMTP11.2* showed preferential expression in flowers, and the remaining members were highly expressed in buds and leaves (**Figure 11C**). During leaf development, the expression of *CsMTP8.1/8.3/10* gradually decreased with leaf maturation, whereas *CsMTP9.1/11.1* displayed the opposite trend. Other members were classified into two groups: genes highly expressed in buds (*CsMTP2/3/7/8.2/11.2*) and genes highly expressed in the first leaf (*CsMTP1/4/5/9.2*) (**Figure 11D**). The promoter regions of *CsMTPs* contained abundant stress-responsive *cis*-elements, which may be closely associated with their induced expression under aluminum, zinc, manganese, and iron stresses (**Supplementary Figure 10**).

In summary, 14 *CsMTP* genes were identified in the tea plant genome and phylogenetically classified into three subfamilies: Zn-CDF, Mn-CDF, and Zn/Fe-CDF, with CsMTP7 as the unique member of the Zn/Fe-CDF subfamily. The majority of CsMTPs were acidic hydrophilic proteins mainly localized to the vacuole; CsMTP8.2 has been confirmed to localize to the cell membrane. The majority of members contained 4–6 TMDs. With the exception of CsMTP5/7, all other CsMTPs contained a conserved cation efflux domain, and marked structural differentiation was observed among subfamilies. *CsMTPs* exhibited distinct tissue- and development-dependent spatiotemporal expression patterns, and their promoters were enriched in stress-responsive *cis*-elements, suggesting that this family plays crucial regulatory roles in metal homeostasis maintenance and stress adaptation in tea plants.

### 3.6 The CsCAX protein family in tea plants

**Figure 12:**
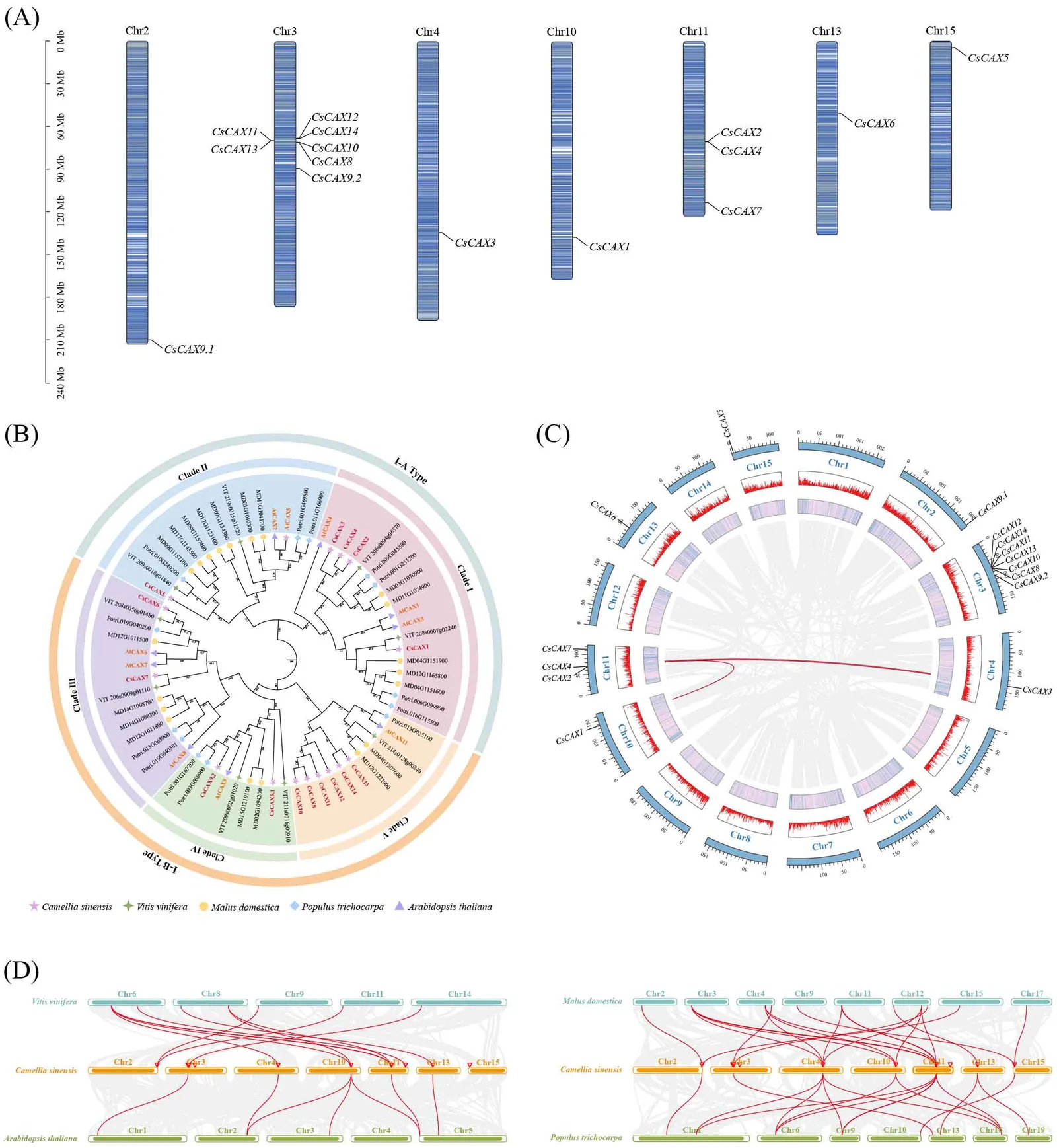
Chromosome distribution, phylogenetic relationship, and synteny analysis of *CsCAX* gene family. **(A)** Chromosomal localization of *CsCAXs* in tea plants. **(B)** Phylogenetic relationship of CAX family proteins from tea plants and other plants. The phylogenetic tree was constructed using the neighbor-joining (NJ) method in MEGA11 with 1000 bootstrap replicates. **(C)** Intraspecies collinearity analysis of *CsCAXs* in tea plants. Gray lines represent all collinear blocks within the tea genome, and red lines highlight duplicated *CsCAXs* gene pairs. **(D)** Interspecies collinearity analysis among tea plant, *Vitis vinifera*, *Arabidopsis*, *Populus trichocarpa*, and *Malus domestica*. Gray lines represent syntenic blocks across genomes, and red lines denote orthologous *CsCAX* relationships.

Cation/H^+^ antiporters (CAXs) belong to the calcium/cation antiporter (CaCA) superfamily and play vital roles in regulating plant calcium homeostasis and cation transport (Ueoka‐Nakanishi et al., 1999). A total of 15 *CsCAX* genes were identified in the tea plant genome and were unevenly distributed across seven chromosomes. Chromosome 3 contained the largest number (seven members), suggesting that it may be the major locus for gene enrichment and expansion of the tea plant CAX family (**Figure 12A**). Phylogenetic analysis classified CsCAXs into five subfamilies (I-V). Subfamily II contained only CsCAX5, whereas Subfamily V included six members (**Figure 12B**). According to the classification of *Arabidopsis* AtCAX subtypes (I-A and I-B) (Shigaki et al., 2006), tea plant Subfamilies I and II belonged to subtype I-A, while Subfamilies III, IV, and V belonged to subtype I-B. Three intraspecific collinear gene pairs were identified in tea plants, all with Ka/Ks values <1, indicating that they may have undergone purifying selection (**Figure 12C**; **Supplementary Table 1**). The number of *CAX* collinear gene pairs between tea plants and woody species (apple, poplar, and grape) was much higher than that between tea plants and *Arabidopsis*, implying that the evolutionary expansion pattern of tea plant CAXs may potentially resemble that of woody plants (**Figure 12D**).

**Figure 13:**
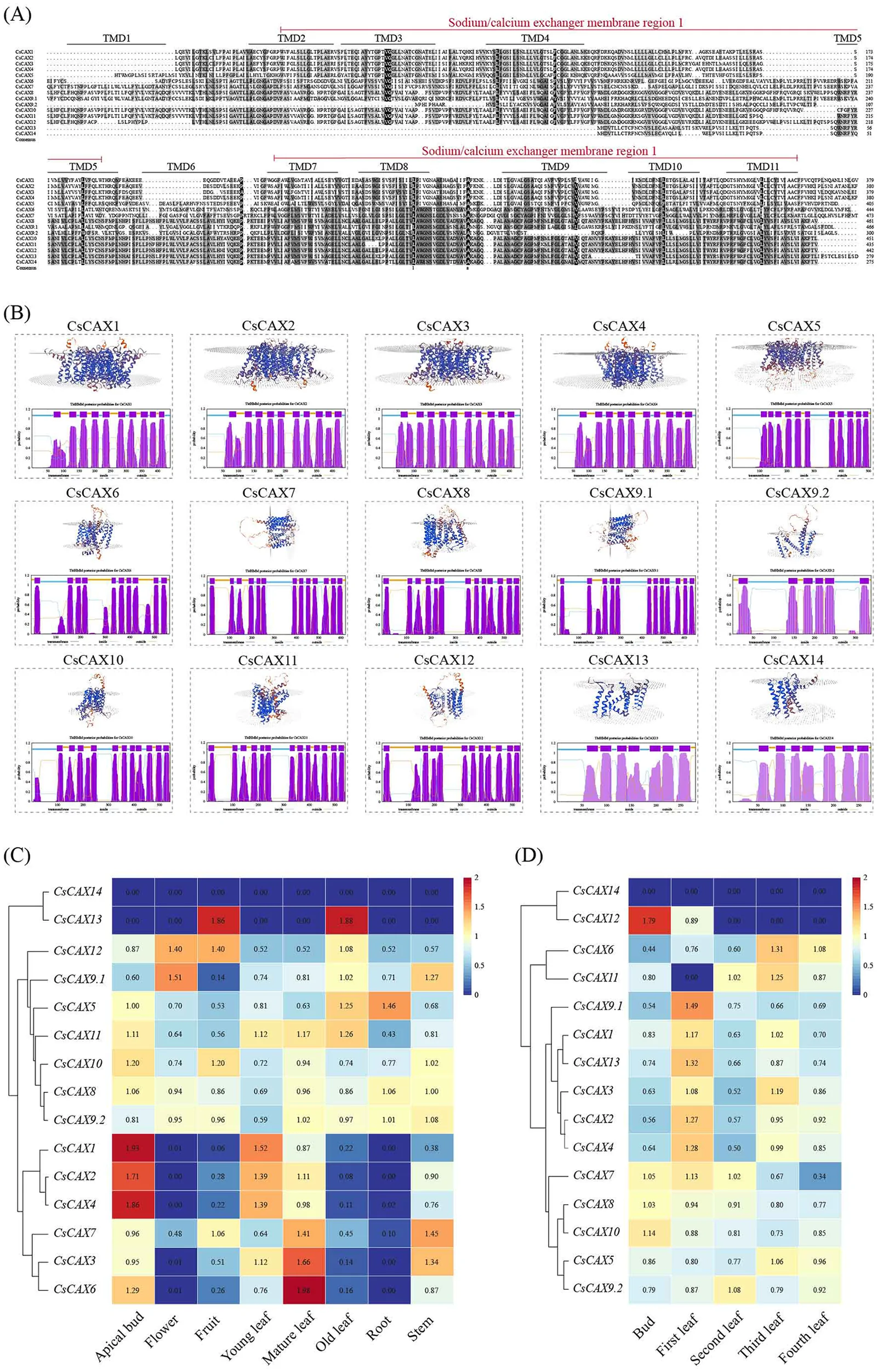
Multiple sequence alignment, three-dimensional structure modeling, transmembrane domain prediction, and tissue-specific expression profiling of CsCAXs in tea plants. **(A)** Multiple sequence alignment analysis of CsCAX proteins in tea plants. The black bars above the sequences denote the TMDs. The red bars above the sequences denote the sodium/calcium exchanger membrane region domain. Alignment coloring: black, ≥75% conservation; dark gray, ≥50% conservation; light gray, ≥33% conservation; dots, gaps. **(B)** Predicted transmembrane domains and three-dimensional structures of CsCAX proteins. The disk-shaped structure composed of gray dots in the three-dimensional protein models represents the membrane. In the transmembrane domain probability plots, purple peaks indicate transmembrane regions, the blue line corresponds to the intracellular compartment, and the yellow line corresponds to the extracellular compartment. **(C)** Heatmap of *CsCAXs* gene expression in apical bud, flower, fruit, young leaf, mature leaf, old leaf, root, and stem. **(D)** Heatmap of *CsCAXs* gene expression in bud and different leaf positions (first to fourth leaf). The hierarchical clustering tree is shown on the left of each heatmap. Expression values are presented as row-wise normalized TPM data..

Tea CsCAX proteins ranged from 275 to 656 aa in length, with MWs of 30.61–72.35 kDa. All CsCAXs were hydrophobic proteins (GRAVY > 0). Subtype I-A members were acidic proteins, while subtype I-B included both acidic and alkaline proteins (**Supplementary Table 2**). Subcellular localization prediction indicated that CsCAXs exhibited subfamily specificity: Subfamilies I and II were localized to the vacuole; Subfamily III was distributed on the cell membrane and Golgi apparatus; Subfamily IV specifically targeted the cell membrane; and Subfamily V was exclusively located in chloroplasts, with several members also showing dual localization to the cell membrane and mitochondria. These divergent localization patterns may suggest functional differentiation in cation transport among subfamilies (**Table 1**). Typical CAX proteins generally contain 11 TMDs, in addition to an N-terminal autoinhibitory region, a calcium domain, and conserved C and D functional domains (Pittman et al., 2004). The majority of CsCAXs in this study contained 10–13 TMDs, while CsCAX9.2/13/14 had fewer (**Table 1**). All CsCAXs contained two highly conserved sodium/calcium exchanger membrane regions, and potentially marked divergence in amino acid sequences and three-dimensional structures was observed between I-A and I-B subtypes (**Figures 13A, B**). The exon number of subtype I-A (10–14) was markedly higher than that of subtype I-B (1–4), which may reflect substantial structural divergence closely related to functional differentiation and expression regulation (**Supplementary Figure 11A**). A total of 10 conserved motifs were detected in CsCAX proteins. Motif 3 was highly conserved in all members, and phylogenetically close members shared similar motif compositions, indicating strong sequence conservation within each subfamily (**Supplementary Figures 11B, C**).

Tissue expression analysis showed that *CsCAX3/6/7* were highly expressed in mature leaves and stems; *CsCAX1/2/4* preferred young tissues such as apical buds and immature leaves; *CsCAX13* was predominantly expressed in fruits and old leaves; *CsCAX14* was barely expressed, and the remaining members showed no significant tissue preference (**Figure 13C**). During leaf development, *CsCAX12* exhibited the highest expression in buds; *CsCAX6/11* were preferentially expressed in the third leaf; *CsCAX1/2/3/4/7/9.1/13* showed high transcript abundance in the first leaf; and other members maintained relatively stable expression levels (**Figure 13D**). *Cis*-element analysis revealed abundant stress- and light-responsive elements in *CsCAX* promoters. Among them, *CsCAX10* contained eight low-temperature-responsive (LTR) elements, suggesting a potential role in cold stress adaptation in tea plants. Notably, the *CsCAX12* promoter was enriched in numerous hormone-related *cis*-elements, including 11 ABA-responsive (ABRE) elements and 16 methyl jasmonate (MeJA)-responsive elements (eight CGTCA-elements and eight TGACG-elements). These findings suggest that *CsCAX12* may modulate metal ion homeostasis and stress tolerance by potentially mediating ABA and MeJA signaling pathways (**Supplementary Figure 12**).

In summary, 15 *CsCAX* genes were identified in the tea plant genome and unevenly distributed on seven chromosomes, with chromosome 3 containing the largest number of members. These CsCAXs were phylogenetically divided into five subfamilies corresponding to *Arabidopsis* I-A (I and II) and I-B (III, IV, and V) subtypes. Intraspecific collinear genes underwent purifying selection, and the *CAX* family in tea plants is evolutionarily closer to those of woody plants. Marked subtype divergence was observed in physicochemical properties, subcellular localization, three-dimensional structure, conserved motifs, and gene structure. Several members displayed tissue- and development-specific expression patterns. *CsCAX* promoters were enriched in stress-, light-, and hormone-responsive elements. In particular, *CsCAX12* contained abundant hormone-related *cis*-elements, implying its involvement in hormone-mediated metal homeostasis and stress responses in tea plants.

## 4 Discussion

### 4.1 From soil fixation to symplastic entry: zinc acquisition strategies in tea plant roots

Roots are the primary organ for plant zinc uptake, with root plasma membrane transporters directly determining acquisition efficiency. In tea plants, *CsZIPs* and *CsNRAMPs* represent the core families mediating transmembrane zinc influx, and their transport properties are highly compatible with the acidophilic trait of tea plants. CsZIP proteins transport Zn^2+^, Fe^2+^, Mn^2+^, and Cu^2+^, and all members participate in intracellular Zn^2+^ homeostasis regulation (Bashir et al., 2021). They show differential responses to zinc stress: *CsZIP1–6* and *CsZIP10–12* are upregulated under zinc deficiency, while high zinc stress induces *CsZIP7/8* transcription and represses *CsZIP2/4* (Singh et al., 2016). Heterologous expression in the yeast *Δzrc1cot1* double mutant further verified the zinc transport and tolerance functions of multiple CsZIP members (Zheng et al., 2022). In this study, the majority of CsZIP proteins localize to the cell membrane and contained a conserved zinc/iron permease domain, acting as key carriers for Zn^2+^ translocation from the apoplast to the symplast. The majority of ZIP proteins function as H^+^/metal symporters dependent on transmembrane proton gradients, making them well adapted to acidic tea plantation soils. A decrease in soil pH from 6.0 to 4.5 increases Zn^2+^ solubility by 100- to 1,000-fold, with available Zn^2+^ concentrations far exceeding the threshold for plant growth (Broadley et al., 2007; Sadeghzadeh and Rengel, 2011). Tea plants actively acidify the rhizosphere via plasma membrane H^+^-ATPases, which mobilize sparingly soluble zinc and maintain proton gradients to drive ZIP-mediated transport (Palmgren, 2001; Santi and Schmidt, 2008). Rhizosphere acidification displays distinct seasonal fluctuations. Enhanced H^+^-ATPase activity during spring bud growth stabilizes Zn^2+^ accumulation in young shoots, which may explain the higher zinc content in spring tea than in summer tea (Wan et al., 2018; Zhao and Zhao, 2019). *CsZIP2/3/4/10/11.1* are highly expressed in tea roots and may efficiently mediate the uptake of mobilized Zn^2+^ using proton gradients from rhizosphere acidification, providing a molecular basis for the superior zinc acquisition capacity of tea plants compared with neutral-soil crops. Additionally, root-secreted citrate and malate form soluble Zn^2+^ chelates that reduce apoplastic precipitation and continuously supply substrates for ZIP transport, synergistically enhancing root zinc uptake efficiency (Morita et al., 2011; Saleem et al., 2022; Chen et al., 2023).

The NRAMP family comprises core divalent metal transporters in plants that recognize substrates including Zn^2+^, Cd^2+^, and Mn^2+^, and plays dual roles in essential mineral homeostasis and heavy metal detoxification (Krämer et al., 2007; Ullah et al., 2018). NRAMP transporters are well characterized in model plants. *Arabidopsis AtNRAMP2* targets the TGN to mediate Golgi Mn^2+^ compartmentation and recycling and also transports Zn^2+^ and Fe^2+^ (Kosuth et al., 2023; Huang et al., 2024). Tonoplast *AtNRAMP3/4* are functionally redundant, exporting vacuolar Mn^2+^/Zn^2+^ to the cytosol and exhibiting high Cd^2+^/Zn^2+^ affinity to regulate cellular metal storage (Huang et al., 2024; Meier et al., 2025). Rice root plasma membrane-located *OsNRAMP5* primarily uptakes Mn^2+^ and Fe^2+^, transports Zn^2+^, and enables long-distance root-to-shoot translocation of these divalent cations (Qu and Nakanishi, 2023; Huang et al., 2026). In this study, CsNRAMP subfamily III was identified as a tea-specific evolutionary clade with strict root-specific expression, suggesting adaptation to acidic plantation habitats. Acidic conditions boost Zn^2+^ availability but also mobilize manganese and aluminum ions, creating multi-ion competitive absorption pressure. We hypothesize that the evolution of this clade refines substrate recognition and multi-metal homeostasis regulation in tea plants, enabling coordinated balance of Zn^2+^, manganese, and iron uptake while avoiding metabolic disorders from ion imbalance. Supporting evidence shows *CsNRAMPs* are differentially expressed under lead stress, suggesting their involvement in lead uptake and translocation (Li et al., 2021). Aluminum stress downregulates *CsNRAMP5* to reduce root manganese uptake and alleviate manganese toxicity (Zhao et al., 2026). Collectively, these findings suggest that the CsNRAMP-mediated metal regulatory network constitutes a core mechanism for tea plants to adapt to acidic stress and maintain multi-metal homeostasis.

### 4.2 Zn transport and partitioning in tea plants

Following root uptake, Zn^2+^ is loaded into xylem vessels for aerial translocation by P1B-type ATPase HMA transporters (Argüello et al., 2007; Huang et al., 2024). HMA functions are well documented: *Arabidopsis AtHMA2/4* loss-of-function causes leaf chlorosis and developmental defects, while *AtHMA4* overexpression enhances Zn^2+^/Cd^2+^ tolerance in yeast (Hussain et al., 2004; Bækgaard et al., 2010). *NtHMA4.1/4.2*, *HvHMAs*, and *AhHMAs* similarly mediate xylem Zn^2+^ loading and shoot Zn^2+^ translocation (Liedschulte et al., 2021; Amini et al., 2022; Li et al., 2024a). In this study, the CsHMA family was classified into two subfamilies with substrate specificities for Zn/Co/Cd/Pb and Cu/Ag. Cell membrane-localized *CsHMA3/5.1/5.3/7*, which were highly expressed in roots and stems, are proposed to pump Zn^2+^ from stelar parenchyma cells into xylem sap, thereby supporting transpiration-driven long-distance transport (Hussain et al., 2004; Sinclair et al., 2007; Wong and Cobbett, 2009; Hennigar and Kelleher, 2012). As perennial woody plants with larger root systems and more complex secondary xylem than herbaceous species, tea plants may rely on CsHMA-mediated active loading to overcome long-distance transport resistance and sustain canopy Zn^2+^ supply (Sato et al., 2011; Yamaji and Ma, 2014). No segmental duplications were detected in the CsHMA family, indicating a conservative expansion pattern, with functional divergence achieved mainly through subfamily substrate specialization, consistent with its role as precise ion pumps.

Phloem-mediated Zn^2+^ remobilization, driven primarily by YSL transporters via metal-nicotianamine (NA) chelate trafficking, is critical for Zn^2+^ delivery to young sink tissues. YSL members exhibit diverse substrate specificities and collectively participate in metal uptake, long-distance translocation, and heavy metal detoxification (Jean et al., 2005). Conserved functions have been verified across species: maize *ZmYSLs* exhibit differential responses to zinc and iron deficiency stresses (Song et al., 2024); *Arabidopsis AtYSL1/3* regulate Zn^2+^ remobilization from senescent leaves to seeds, *AtYSL2* transports Fe^2+^/Cu^2+^/Zn^2+^ to maintain metal homeostasis and alleviate toxicity, and the *ysl1ysl3* double mutant shows reduced seed metal accumulation (DiDonato et al., 2004; Schaaf et al., 2005; Chu et al., 2010); barley *HvYSL2* sustains root metal homeostasis under Fe^2+^ deficiency (Araki et al., 2011). In tea plants, *CsYSL* genes show distinct tissue specificity: root-specific *CsYSL1.2/2* may mediate metal chelate transmembrane transport, whereas leaf-abundant *CsYSL6/8* and maturation-induced *CsYSL1.2/2/5* may support Zn^2+^ mobilization during leaf development. This pattern aligns with the source-sink paradigm: mature leaves (Zn storage sources) load Zn-NA complexes into the phloem via CsYSLs for delivery to high-demand sink tissues such as apical buds and young leaves. During spring flushing, when young shoot Zn^2+^ demand surges, upregulated *CsYSL* expression in mature leaves accelerates phloem Zn^2+^ remobilization to growing shoots, providing a molecular explanation for the higher Zn^2+^ content in spring tea than in summer tea.

### 4.3 Cellular compartmentalization and detoxification

Subcellular compartmentalization is central to cytosolic Zn^2+^ homeostasis and metal detoxification in plants, with vacuolar Zn^2+^ sequestration mediated by the MTP family (Kobae et al., 2004; Kawachi et al., 2012). In *Arabidopsis*, *AtMTP1–3* drive vacuolar Zn^2+^ detoxification, *AtMTP2* modulates root-to-shoot Zn^2+^ translocation under deficiency, and *AtMTP5/12* form a Golgi-localized complex for organellar metal homeostasis (Arrivault et al., 2006; Kawachi et al., 2008; Fujiwara et al., 2015; Sinclair et al., 2018). In tea plants, the majority of CsMTP proteins localize to the tonoplast. The Zn-CDF subfamily (CsMTP1-4), harboring a conserved cation efflux domain, functions as an H^+^/Zn^2+^ antiporter that pumps excess cytosolic Zn^2+^ into the vacuole. This mechanism is critical for tea plants growing in acidic soils with high Zn^2+^ bioavailability: vacuolar compartmentalization rapidly reduces free cytosolic Zn^2+^ to avert cytotoxicity and releases stored Zn^2+^ under deficiency, acting as a homeostatic buffer (Clemens, 2006; Lin and Aarts, 2012). CsMTP1/2/3/4 are highly expressed in roots and stems and may serve as the first line of Zn^2+^ detoxification, while shoot-abundant members may maintain Zn^2+^ homeostasis in photosynthetic tissues, consistent with tissue-specific Zn^2+^ exposure and metabolic demands. Previous studies have confirmed the multi-metal roles of CsMTPs: *CsMTP11* specifically regulates Mn^2+^ homeostasis (Yuan et al., 2017); *CsMTP8* overexpression enhances high-Mn^2+^ tolerance (Li et al., 2017); *CsMTP8.2* effluxes excess Mn^2+^ to alleviate toxicity, as demonstrated in heterologous yeast and *Arabidopsis* systems (Zhang et al., 2020); *CsMTP1* exhibits Zn^2+^ efflux activity and partially rescues the zinc-sensitive phenotype of the yeast *Δzrc1cot1* mutant (Zhang et al., 2019); and *CsMTP4* was recently reported to exert dual functions in both root Zn uptake and Zn excess detoxification in tea plants (Li et al., 2024b).

Beyond the MTP family, CAX transporters also contribute to vacuolar Zn^2+^ sequestration and detoxification. With high transport capacity and low substrate affinity, CAX proteins recognize diverse divalent cations (Ca^2+^, Zn^2+^, and Mn^2+^) and function as central hubs integrating ion homeostasis, stress signaling, and nutrient storage (Pittman and Hirschi, 2024). In *Arabidopsis*, *AtCAX2/4* sequester cytosolic Cd^2+^, Zn^2+^, and Mn^2+^ into vacuoles to reduce cytotoxicity (Koren’kov et al., 2006), while *AtCAX1/3* promote Mn^2+^ and Zn^2+^ accumulation in stems at the expense of Ca^2+^ and Mg^2+^ (Cheng et al., 2005). The tea plant CsCAX subfamilies show distinct subcellular localization (tonoplast, plasma membrane, Golgi apparatus, and chloroplast) with clear functional partitioning. Tonoplast-localized subfamilies I and II synergize with CsMTPs to enhance vacuolar Zn sequestration via cation/H^+^ exchange. The chloroplast-localized subfamily V delivers Zn into the stroma to supply cofactors for key metalloenzymes (including carbonic anhydrase and Cu/Zn-SOD) and sustain photosynthetic metabolism (López-Millán et al., 2004; Podar et al., 2012). This dual “detoxification-supply” mode is functionally essential: under Zn^2+^ excess, vacuolar MTP and CAX jointly sequester toxic ions, whereas under normal Zn^2+^ supply, organellar CAX and ZIP mediate precise Zn^2+^ allocation to balance stress tolerance with basal physiological function.

### 4.4 Crosstalk between zinc homeostasis and metabolic integration

Six major zinc transporter families (ZIP, NRAMP, HMA, YSL, MTP, and CAX) collectively establish a sophisticated Zn homeostasis network in tea plants, which tightly couples carbon metabolism with secondary metabolite biosynthesis. Root CsZIPs prioritize Zn^2+^ acquisition via nicotianamine chelation in acidic tea soils (pH 4.0–5.5), thereby alleviating competitive antagonism by Mn^2+^, Fe^2+^, and Al³^+^. CsHMAs mediate active xylem Zn^2+^ loading to sustain continuous Zn^2+^ delivery for carbonic anhydrase-dependent carbon fixation in photosynthetic tissues. Mature leaf-enriched CsYSLs facilitate phloem-based translocation of Zn-nicotianamine complexes, providing essential cofactors for PAL and C4H and supporting catechin accumulation during spring shoot development (Wang et al., 2012). Recent work further demonstrated that zinc directly promotes trihydroxylated catechin biosynthesis via the zinc-responsive protein CsHIPP3, which interacts with CsF3′5′H1 to enhance flavonoid hydroxylation (Hu et al., 2024). Tonoplast-localized CsMTPs and CsCAXs function as a Zn^2+^ buffering system to sequester excess Zn^2+^ and reduce oxidative stress. Under high-light conditions, preferential Zn^2+^ allocation to Cu/Zn-SOD for antioxidant defense rewires carbon flux toward soluble carbohydrates rather than flavonoids, explaining the seasonal decline in summer tea quality. These transporter genes exhibit tissue- and development-specific expression, with higher transcript abundance in young shoots. Their promoters harbor diverse stress- and hormone-responsive cis-elements, enabling environmental signals to reshape Zn^2+^ distribution and dynamically modulate the biosynthesis of polyphenols, theanine, and caffeine.

## 5 Conclusions and limitations

**Figure 14:**
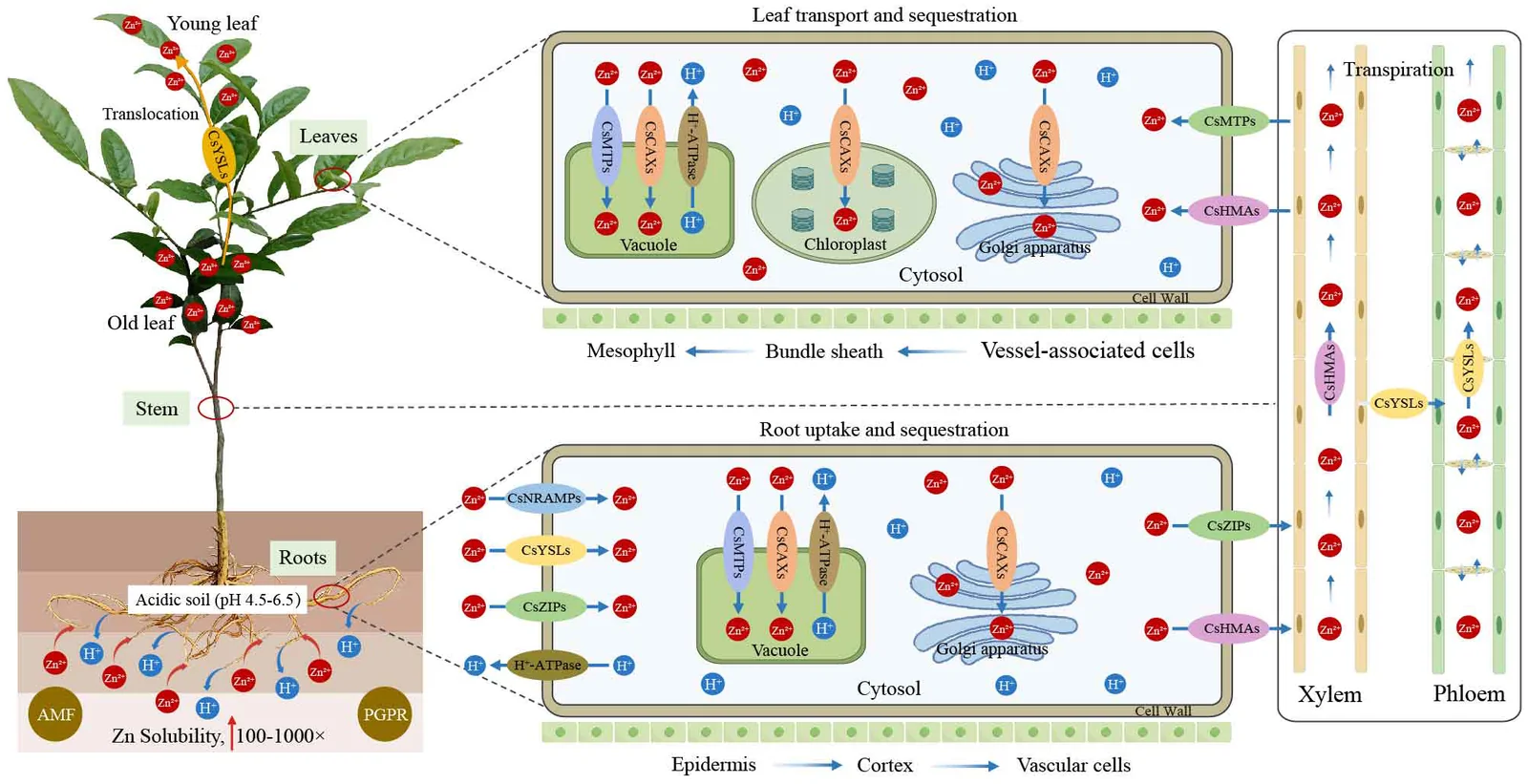
A schematic diagram of the mechanisms of zinc uptake, translocation, and detoxification in tea plants. (1) Rhizosphere acquisition: Tea roots mobilize sparingly soluble soil Zn via plasma membrane H^+^-ATPase-mediated acidification, organic acid (malate/citrate) exudation, and symbiosis with arbuscular mycorrhizal fungi (AMF) and plant growth-promoting rhizobacteria (PGPR). CsZIPs and CsNRAMPs primarily mediate Zn^2+^ influx from the apoplast into the cytosol, dependent on the H^+^ electrochemical gradient; (2) Tissue distribution: Root-to-shoot translocation is mediated by CsHMAs (P1B-type ATPases) via xylem loading; CsZIPs (putative Zn^2+^/H^+^ symporters) and CsYSLs (metal-nicotianamine complex transporter) facilitate phloem remobilization and source-to-sink redistribution, with CsYSLs specifically remobilizing Zn from mature to young leaves. CsCAXs additionally mediate Zn allocation from the cytosol to chloroplasts and other organelles, ensuring photosynthetic apparatus function; (3) Intracellular buffering: CsMTPs mediate vacuolar sequestration via H^+^/Zn^2+^ antiport, energized by the trans-tonoplast proton gradient generated by V-ATPase; CsCAXs exhibit dual functionality in both vacuolar compartmentalization and organellar (chloroplast) Zn distribution, cooperatively maintaining intracellular Zn homeostasis.

A sophisticated zinc homeostasis regulatory system composed of “acquisition-distribution-buffering” modules has been identified in tea plants. During zinc acquisition, root transporters CsZIPs, CsYSLs, and CsNRAMPs cooperate with cell membrane H^+^-ATPase-induced rhizosphere acidification. Combined with symbiotic associations with AMF and PGPR, tea roots efficiently take up zinc from the soil. For internal distribution, P1B-type ATPase CsHMAs are responsible for xylem zinc loading, whereas phloem-localized CsYSLs regulate long-distance zinc translocation toward metabolically vigorous sink tissues. In the buffering and detoxification module, tonoplast-localized CsMTPs and CsCAXs coordinate with the apoplastic oxalate precipitation pathway to sequester zinc, enabling tea plants to simultaneously resist both zinc deficiency and excess zinc toxicity (**Figure 14**).

Objectively, the zinc transport and homeostasis regulatory models proposed in this study are inferred from genome-wide identification, sequence homology analysis, and published evidence, without functional validation via tea plant transgenesis, heterologous functional complementation, or other in planta assays. The reported gene regulatory patterns, ion transport pathways, and hormone crosstalk networks are exclusively bioinformatic predictions with no experimental confirmation. *Cis*-element enrichment and phylogenetic clustering provide only preliminary clues regarding gene function and do not constitute conclusive evidence. Further functional assays based on tea genetic transformation systems, including gene overexpression and knockout, are required to delineate the authentic physiological roles of these transporters in mediating zinc uptake, long-distance translocation, and vacuolar sequestration.

Ultimately, zinc is not merely a static micronutrient but also a functional signaling ion that coordinately regulates secondary metabolism, organ differentiation, and stress tolerance throughout the entire life cycle of perennial plants. The zinc homeostasis network in tea plants offers a paradigm for deciphering the coordinated regulatory mechanisms linking nutritional signals to growth and developmental programs in perennial woody species. As anthropogenic climate change intensifies, soil acidification and heavy metal stress have become growing challenges. The zinc homeostasis pathways in tea plants, shaped by long-term adaptation to acidic mountain soils in Asia, provide valuable genetic targets for improving stress tolerance in temperate and tropical crops.

## Data Availability

The original contributions presented in the study are included in the article/**Supplementary Material**, further inquiries can be directed to the corresponding author/s.
