# Supplementary material for: Systematic mining and characterization of metal transporter families regulating zinc homeostasis provide insights into metal homeostasis in Camellia sinensis
Source: Front Plant Sci. 2026 Aug 18;17:1901777. doi: 10.3389/fpls.2026.1901777 (PMC13529523; doi:10.3389/fpls.2026.1901777)
Supplement: Supplementary file 1 [file SupplementaryFile1.docx]

Supplementary Material

# **Supplementary Tables**

## Supplementary Table 1 The Ka/Ks ratios of *CsZIPs*, *CsNRAMPs*, *CsYSLs*, *CsMTPs* and *CsCAXs* collinearity gene pairs in tea plant.

| Sequence 1 | Sequence 2 | Ka | Ks | Ka/Ks | EffectiveLen | AverageS-sites | AverageN-sites | cN | cS | pN | pS |
| --- | --- | --- | --- | --- | --- | --- | --- | --- | --- | --- | --- |
| *CsZIP6.1* | *CsZIP6.2* | 0.071 | 0.467 | 0.152 | 990 | 242 | 748 | 50.83 | 84.17 | 0.068 | 0.348 |
| *CsNRAMP2* | *CsNRAMP3.2* | 0.163 | 1.379 | 0.119 | 1530 | 368.67 | 1161.33 | 170.5 | 232.5 | 0.147 | 0.631 |
| *CsNRAMP3.2* | *CsNRAMP4* | 0.089 | 0.649 | 0.138 | 1542 | 374.33 | 1167.67 | 98.5 | 162.5 | 0.084 | 0.434 |
| *CsNRAMP7* | *CsNRAMP8* | 0.110 | 0.577 | 0.191 | 1548 | 370.5 | 1177.5 | 120.83 | 149.17 | 0.103 | 0.403 |
| *CsYSL1.1* | *CsYSL1.2* | 0.131 | 0.700 | 0.187 | 1908 | 449.67 | 1458.33 | 175.33 | 204.67 | 0.120 | 0.455 |
| *CsYSL1.1* | *CsYSL2* | 0.172 | 1.849 | 0.093 | 1968 | 468.83 | 1499.17 | 230.25 | 321.75 | 0.154 | 0.686 |
| *CsYSL2* | *CsYSL3* | 0.068 | 0.521 | 0.130 | 1986 | 471.5 | 1514.5 | 97.92 | 177.08 | 0.065 | 0.376 |
| *CsYSL4* | *CsYSL6* | 0.886 | 0.387 | 0.229 | 1731 | 422.5 | 1308.5 | 109.33 | 127.67 | 0.084 | 0.302 |
| *CsMTP1* | *CsMTP2* | 0.087 | 0.410 | 0.214 | 1107 | 254.67 | 852.33 | 70.5 | 80.5 | 0.083 | 0.316 |
| *CsMTP9.1* | *CsMTP10* | 0.132 | 0.360 | 0.366 | 1173 | 269.25 | 903.75 | 109.08 | 76.92 | 0.121 | 0.286 |
| *CsMTP11.1* | *CsMTP11.2* | 0.080 | 0.464 | 0.173 | 855 | 207.25 | 647.75 | 49.33 | 71.67 | 0.076 | 0.346 |
| *CsCAX1* | *CsCAX2* | 0.177 | 1.389 | 0.128 | 1320 | 321.33 | 998.67 | 157.83 | 203.17 | 0.158 | 0.632 |
| *CsCAX2* | *CsCAX3* | 0.104 | 0.559 | 0.185 | 1287 | 311.42 | 975.58 | 94.33 | 122.67 | 0.097 | 0.394 |
| *CsCAX3* | *CsCAX4* | 0.104 | 0.550 | 0.189 | 1287 | 310.92 | 976.08 | 94.83 | 121.17 | 0.097 | 0.390 |

## Supplementary Table 2 The Basic information of analysis of *CsZIPs*, *CsNRAMPs*, *CsHMAs*, *CsYSLs*, *CsMTPs* and *CsCAXs* family in tea plant

| Gene family | Gene Name | Gene ID | CDS length | Protein length | Molecular weight (KDa) | Isoelectric point (pI) | Grand average of hydropathicity |
| --- | --- | --- | --- | --- | --- | --- | --- |
| *CsZIPs* | *CsZIP1* | CSS0029626 | 1056 bp | 351 aa | 37.50 | 8.21 | 0.434 |
|  | *CsZIP2* | CSS0004796 | 1026 bp | 341 aa | 36.76 | 7.68 | 0.497 |
|  | *CsZIP3* | CSS0022695 | 1059 bp | 352 aa | 37.11 | 5.98 | 0.540 |
|  | *CsZIP4* | CSS0043874 | 1128 bp | 375 aa | 39.67 | 5.64 | 0.417 |
|  | *CsZIP5* | CSS0010396 | 1083 bp | 360 aa | 37.43 | 6.88 | 0.637 |
|  | *CsZIP6.1* | CSS0007950 | 1017 bp | 338 aa | 35.71 | 5.69 | 0.657 |
|  | *CsZIP6.2* | CSS0048283 | 1005 bp | 334 aa | 35.74 | 6.05 | 0.634 |
|  | *CsZIP7* | CSS0027771 | 1050 bp | 349 aa | 36.74 | 8.51 | 0.682 |
|  | *CsZIP10* | CSS0036144 | 1047 bp | 348 aa | 37.12 | 5.76 | 0.728 |
|  | *CsZIP11.1* | CSS0011926 | 1014 bp | 337 aa | 36.05 | 6.21 | 0.620 |
|  | *CsZIP11.2* | CSS0008965 | 1044 bp | 347 aa | 37.62 | 6.81 | 0.702 |
|  | *CsZIP12.1* | CSS0045198 | 1038 bp | 345 aa | 35.91 | 8.17 | 0.628 |
|  | *CsZIP12.2* | CSS0017488 | 1089 bp | 362 aa | 37.63 | 6.37 | 0.642 |
| *CsNRAMPs* | *CsNRAMP1* | CSS0031499 | 1551 bp | 516 aa | 55.91 | 8.90 | 0.666 |
|  | *CsNRAMP2* | CSS0044220 | 1605 bp | 534 aa | 58.26 | 4.89 | 0.523 |
|  | *CsNRAMP3.1* | CSS0041547 | 1368 bp | 455 aa | 50.36 | 8.33 | 0.636 |
|  | *CsNRAMP3.2* | CSS0022297 | 1569 bp | 522 aa | 57.60 | 4.94 | 0.522 |
|  | *CsNRAMP3.3* | CSS0047249 | 1251 bp | 416 aa | 45.70 | 8.44 | 0.726 |
|  | *CsNRAMP4* | CSS0021034 | 1545 bp | 514 aa | 56.27 | 5.12 | 0.636 |
|  | *CsNRAMP5* | CSS0004419 | 1578 bp | 525 aa | 57.41 | 5.23 | 0.487 |
|  | *CsNRAMP6* | CSS0042984 | 1428 bp | 475 aa | 51.52 | 9.22 | 0.714 |
|  | *CsNRAMP7* | CSS0046035 | 1554 bp | 517 aa | 56.25 | 8.99 | 0.697 |
|  | *CsNRAMP8* | CSS0019007 | 1668 bp | 555 aa | 60.64 | 8.70 | 0.494 |
|  | *CsNRAMP9* | CSS0007121 | 1635 bp | 544 aa | 59 59 | 8.50 | 0.568 |
|  | *CsNRAMP10* | CSS0046206 | 1575 bp | 524 aa | 56.84 | 8.89 | 0.678 |
| *CsHMAs* | *CsHMA1* | CSS0024485 | 2478 bp | 825 aa | 89.96 | 8.72 | 0.093 |
|  | *CsHMA3* | CSS0005701 | 2703 bp | 900 aa | 97.24 | 7.51 | 0.014 |
|  | *CsHMA5.1* | CSS0042325 | 2967 bp | 988 aa | 107.48 | 5.69 | 0.123 |
|  | *CsHMA5.2* | CSS0024211 | 2340 bp | 779 aa | 85.19 | 6.61 | 0.177 |
|  | *CsHMA5.3* | CSS0027458 | 2877 bp | 958 aa | 104.46 | 5.10 | 0.127 |
|  | *CsHMA5.4* | CSS0023767 | 2814 bp | 937 aa | 105.21 | 5.41 | 0.167 |
|  | *CsHMA6.1* | CSS0044959 | 2841 bp | 946 aa | 100.19 | 8.82 | 0.122 |
|  | *CsHMA6.2* | CSS0014897 | 531 bp | 176 aa | 18.00 | 8.90 | 0.069 |
|  | *CsHMA7* | CSS0014840 | 3027 bp | 1008 aa | 107.83 | 5.21 | 0.275 |
|  | *CsHMA8* | CSS0040015 | 2646 bp | 881 aa | 93.33 | 5.57 | 0.244 |
| *CsYSLs* | *CsYSL1.1* | CSS0007085 | 2007 bp | 668 aa | 73.29 | 9.11 | 0.535 |
|  | *CsYSL1.2* | CSS0025418 | 1965 bp | 654 aa | 71.92 | 8.99 | 0.462 |
|  | *CsYSL2* | CSS0039421 | 1998 bp | 665 aa | 73.28 | 8.98 | 0.484 |
|  | *CsYSL3* | CSS0025120 | 2055 bp | 684 aa | 75.25 | 8.97 | 0.493 |
|  | *CsYSL4* | CSS0026851 | 2046 bp | 681 aa | 74.72 | 5.36 | 0.479 |
|  | *CsYSL5* | CSS0007763 | 2133 bp | 710 aa | 78.25 | 8.99 | 0.371 |
|  | *CsYSL6* | CSS0018457 | 1956 bp | 651 aa | 70.42 | 6.85 | 0.624 |
|  | *CsYSL7* | CSS0025441 | 2022 bp | 673 aa | 74.04 | 9.04 | 0.494 |
|  | *CsYSL8* | CSS0044292 | 1089 bp | 362 aa | 40.45 | 9.12 | 0.419 |
|  | *CsYSL9* | CSS0047696 | 2301 bp | 766 aa | 83.46 | 8.62 | 0.433 |
| *CsMTPs* | *CsMTP1* | CSS0036317 | 1353 bp | 450 aa | 49.26 | 5.94 | 0.049 |
|  | *CsMTP2* | CSS0014621 | 1122 bp | 373 aa | 41.21 | 6.23 | 0.275 |
|  | *CsMTP3* | CSS0049997 | 1254 bp | 417 aa | 46.21 | 6.10 | 0.167 |
|  | *CsMTP4* | CSS0028321 | 1296 bp | 431 aa | 47.24 | 5.51 | 0.170 |
|  | *CsMTP5* | CSS0003318 | 1182 bp | 393 aa | 44.30 | 8.75 | 0.089 |
|  | *CsMTP7* | CSS0006652 | 1452 bp | 483 aa | 52.81 | 9.12 | 0.004 |
|  | *CsMTP8.1* | CSS0043434 | 1218 bp | 405 aa | 45.75 | 5.10 | 0.092 |
|  | *CsMTP8.2* | CSS0042503 | 1239 bp | 412 aa | 46.29 | 5.00 | -0.013 |
|  | *CsMTP8.3* | CSS0033190 | 312 bp | 103 aa | 11.40 | 5.18 | 0.416 |
|  | *CsMTP9.1* | CSS0044030 | 1251 bp | 416 aa | 47.66 | 6.96 | -0.187 |
|  | *CsMTP9.2* | CSS0037602 | 633 bp | 210 aa | 24.17 | 6.30 | 0.112 |
|  | *CsMTP10* | CSS0023067 | 1179 bp | 392 aa | 44.78 | 6.72 | -0.044 |
|  | *CsMTP11.1* | CSS0026766 | 1200 bp | 399 aa | 45.12 | 5.21 | 0.072 |
|  | *CsMTP11.2* | CSS0018656 | 858 bp | 285 aa | 32.26 | 5.91 | 0.356 |
| *CsCAXs* | *CsCAX1* | CSS0036754 | 1329 bp | 442 aa | 47.79 | 5.85 | 0.648 |
|  | *CsCAX2* | CSS0007722 | 1332 bp | 443 aa | 48.49 | 5.76 | 0.663 |
|  | *CsCAX3* | CSS0006886 | 1311 bp | 436 aa | 47.75 | 6.14 | 0.690 |
|  | *CsCAX4* | CSS0034222 | 1332 bp | 443 aa | 48.51 | 5.76 | 0.663 |
|  | *CsCAX5* | CSS0000893 | 1533 bp | 510 aa | 56.24 | 5.30 | 0.455 |
|  | *CsCAX6* | CSS0026495 | 1692 bp | 563 aa | 62.06 | 8.24 | 0.535 |
|  | *CsCAX7* | CSS0007913 | 1869 bp | 622 aa | 67.44 | 6.83 | 0.672 |
|  | *CsCAX8* | CSS0002251 | 1707 bp | 568 aa | 62.02 | 8.45 | 0.778 |
|  | *CsCAX9.1* | CSS0006324 | 1971 bp | 656 aa | 72.35 | 5.66 | 0.538 |
|  | *CsCAX9.2* | CSS0015661 | 1023 bp | 340 aa | 37.44 | 8.71 | 0.659 |
|  | *CsCAX10* | CSS0049964 | 1635 bp | 544 aa | 59.65 | 8.33 | 0.769 |
|  | *CsCAX11* | CSS0031507 | 1632 bp | 543 aa | 59.46 | 8.20 | 0.883 |
|  | *CsCAX12* | CSS0018394 | 1602 bp | 533 aa | 58.36 | 6.69 | 0.764 |
|  | *CsCAX13* | CSS0020087 | 846 bp | 281 aa | 30.71 | 6.85 | 1.042 |
|  | *CsCAX14* | CSS0024397 | 828 bp | 275 aa | 30.61 | 6.98 | 0.939 |

# **Supplementary Figures**

## **Supplementary Figure 1** Gene structure analysis of *CsZIPs* genes and the conserved motifs of CsZIPs proteins in tea plant. **(A)** Gene structure analysis of *CsZIPs*. Blue boxes and black lines represent coding sequences and introns, respectively. **(B)** MEME analysis of the distribution of conserved motifs in CsZIPs. Different motifs are represented by the colors. **(C)** Motif sequences and consensus.

## **Supplementary Figure 2** Analysis of *cis*-acting regulatory elements in *CsZIPs* promoters. **(A)** Distribution and classification of cis-elements within the 2.0 kb upstream regions of *CsZIPs* genes. Different colors indicate distinct functional categories. **(B)** Quantity of *cis*-acting elements in the *CsZIPs* promoters. The heatmap displays the frequency of each element type, with color intensity representing copy numbers.

## **Supplementary Figure 3** Gene structure analysis of *CsNRAMPs* genes and the conserved motifs of CsNRAMPs proteins in tea plant. **(A)** Gene structure analysis of *CsNRAMPs*. Blue boxes and black lines represent coding sequences and introns, respectively. **(B)** MEME analysis of the distribution of conserved motifs in CsNRAMPs. Different motifs are represented by the colors. **(C)** Motif sequences and consensus.

## **Supplementary Figure 4** Analysis of *cis*-acting regulatory elements in *CsNRAMPs* promoters. **(A)** Distribution and classification of cis-elements within the 2.0 kb upstream regions of *CsNRAMPs* genes. Different colors indicate distinct functional categories. **(B)** Quantity of *cis*-acting elements in the *CsNRAMPs* promoters. The heatmap displays the frequency of each element type, with color intensity representing copy numbers.

## **Supplementary Figure 5** Gene structure analysis of *CsHMAs* genes and the conserved motifs of CsHMAs proteins in tea plant. **(A)** Gene structure analysis of *CsHMAs*. Blue boxes and black lines represent coding sequences and introns, respectively. **(B)** MEME analysis of the distribution of conserved motifs in CsHMAs. Different motifs are represented by the colors. **(C)** Motif sequences and consensus.

## **Supplementary Figure 6** Analysis of *cis*-acting regulatory elements in *CsHMAs* promoters. **(A)** Distribution and classification of cis-elements within the 2.0 kb upstream regions of *CsHMAs* genes. Different colors indicate distinct functional categories. **(B)** Quantity of *cis*-acting elements in the *CsHMAs* promoters. The heatmap displays the frequency of each element type, with color intensity representing copy numbers.

## **Supplementary Figure 7** Gene structure analysis of *CsYSLs* genes and the conserved motifs of CsYSLs proteins in tea plant. **(A)** Gene structure analysis of CsYSLs. Blue boxes and black lines represent coding sequences and introns, respectively. **(B)** MEME analysis of the distribution of conserved motifs in CsYSLs. Different motifs are represented by the colors. **(C)** Motif sequences and consensus.

## **Supplementary Figure 8** Analysis of *cis*-acting regulatory elements in *CsYSLs* promoters. **(A)** Distribution and classification of cis-elements within the 2.0 kb upstream regions of *CsYSLs* genes. Different colors indicate distinct functional categories. **(B)** Quantity of *cis*-acting elements in the *CsYSLs* promoters. The heatmap displays the frequency of each element type, with color intensity representing copy numbers.

## **Supplementary Figure 9** Gene structure analysis of *CsMTPs* genes and the conserved motifs of CsMTPs proteins in tea plant. **(A)** Gene structure analysis of *CsMTPs*. Blue boxes and black lines represent coding sequences and introns, respectively. **(B)** MEME analysis of the distribution of conserved motifs in CsMTPs. Different motifs are represented by the colors. **(C)** Motif sequences and consensus.

## **Supplementary Figure 10** Analysis of *cis*-acting regulatory elements in *CsMTPs* promoters. **(A)** Distribution and classification of cis-elements within the 2.0 kb upstream regions of *CsMTPs* genes. Different colors indicate distinct functional categories. **(B)** Quantity of *cis*-acting elements in the *CsMTPs* promoters. The heatmap displays the frequency of each element type, with color intensity representing copy numbers.

## **Supplementary Figure 11** Gene structure analysis of *CsCAXs* genes and the conserved motifs of CsCAXs proteins in tea plant. **(A)** Gene structure analysis of *CsCAXs*. Blue boxes and black lines represent coding sequences and introns, respectively. **(B)** MEME analysis of the distribution of conserved motifs in CsCAXs. Different motifs are represented by the colors. **(C)** Motif sequences and consensus.

## **Supplementary Figure 12** Analysis of *cis*-acting regulatory elements in *CsCAXs* promoters. **(A)** Distribution and classification of cis-elements within the 2.0 kb upstream regions of *CsCAXs* genes. Different colors indicate distinct functional categories. **(B)** Quantity of *cis*-acting elements in the *CsCAXs* promoters. The heatmap displays the frequency of each element type, with color intensity representing copy numbers.
